# Genetically Matched Human iPS Cells Reveal that Propensity for Cartilage and Bone Differentiation Differs with Clones, not Cell Type of Origin

**DOI:** 10.1371/journal.pone.0053771

**Published:** 2013-01-31

**Authors:** Akira Nasu, Makoto Ikeya, Takuya Yamamoto, Akira Watanabe, Yonghui Jin, Yoshihisa Matsumoto, Kazuo Hayakawa, Naoki Amano, Shingo Sato, Kenji Osafune, Tomoki Aoyama, Takashi Nakamura, Tomohisa Kato, Junya Toguchida

**Affiliations:** 1 Department of Tissue Regeneration, Institute for Frontier Medical Sciences, Kyoto University, Kyoto, Japan; 2 Department of Orthopaedic Surgery, Graduate School of Medicine, Kyoto University, Kyoto, Japan; 3 Department of Cell Growth and Differentiation, Center for iPS Cell Research and Application, Kyoto University, Kyoto, Japan; 4 Department of Reprogramming Science, Center for iPS Cell Research and Application, Kyoto University, Kyoto, Japan; 5 Department of Orthopaedic Surgery, Graduate School of Medical Sciences, Nagoya City University, Nagoya, Japan; National Institute of Environmental Health Sciences, United States of America

## Abstract

**Background:**

For regenerative therapy using induced pluripotent stem cell (iPSC) technology, cell type of origin to be reprogrammed should be chosen based on accessibility and reprogramming efficiency. Some studies report that iPSCs exhibited a preference for differentiation into their original cell lineages, while others did not. Therefore, the type of cell which is most appropriate as a source for iPSCs needs to be clarified.

**Methodology/Principal Findings:**

Genetically matched human iPSCs from different origins were generated using bone marrow stromal cells (BMSCs) and dermal fibroblasts (DFs) of the same donor, and global gene expression profile, DNA methylation status, and differentiation properties into the chondrogenic and osteogenic lineage of each clone were analyzed. Although genome-wide profiling of DNA methylation suggested tissue memory in iPSCs, genes expressed differentially in BMSCs and DFs were equally silenced in our bona fide iPSCs. After cell-autonomous and induced differentiation, each iPSC clone exhibited various differentiation properties, which did not correlate with cell-of-origin.

**Conclusions/Significance:**

The reprogramming process may remove the difference between DFs and BMSCs at least for chondrogenic and osteogenic differentiation. Qualified and genetically matched human iPSC clone sets established in this study are valuable resources for further basic study of clonal differences.

## Introduction

The establishment of induced pluripotent stem cells (iPSCs) has had a profound impact on both basic biology and clinical medicine. iPSCs were first generated in mice by Takahashi and Yamanaka in 2006 [1], where mouse somatic cells were reprogrammed into pluripotent embryonic stem cell (ESC)-like cells through retroviral infection of four transcription factors, Oct3/4, Sox2, Klf4, and c-Myc. These cells closely resemble ESCs in terms of the expression of pluripotency-associated genes, differentiation in vitro into three germ layers, formation of teratomas in vivo, contribution to chimeras, and transmission into germ lines [1], [2]. Subsequently, several groups reported the successful generation of human iPSCs using similar strategies [3], [4], [5]. Because of their infinite proliferative ability and pluripotent differentiation properties, human iPSCs have been regarded as a promising source for cell-based regenerative therapy. Avoiding ethical issues related to the use of fertilized eggs is an advantage of iPSCs, and the application of HLA-matched iPSCs may be able to minimize adjuvant immunosuppressive therapy after transplantation.

An issue related to cell therapy using iPSCs is whether the type of original somatic cell has any influence on the properties of the iPSCs generated from it. It has been shown that iPSCs can be generated from a wide variety of cells [3], [6], [7], [8], [9], [10], [11], [12]. The efficiency of iPSC generation differs, and it is difficult to determine the best cell type, because efficiency also differs with the reprogramming method. The difference in differentiation properties is a more serious issue than efficiency. Blood cells are one of the most promising sources for reprogramming because they can be obtained with minimal invasion and the reprogramming efficiency is sufficient [12], [13]. It has been reported that blood-derived low-passage mouse iPSCs were less able to differentiate into osteoblasts than mouse ESCs and fibroblast-derived iPSCs [14], and that blood-derived low-passage human iPSCs were less able to differentiate into keratinocytes than human ESCs [15], which suggests that blood-derived iPSCs are not an appropriate source for bone or skin regeneration.

Bone marrow stromal cells (BMSCs) include mesenchymal stem cells (MSCs), which are tissue stem cells able to differentiate into multiple cell types in mesenchymal tissues such as chondrocytes, osteoblasts, and adipocytes [16], [17], [18]. The differentiation properties of MSCs into non-mesodermal cells such as neuronal cells and hepatocytes have also been demonstrated [19], [20]. Because MSCs can be obtained from bone marrow or adipose tissue by relatively simple methods, their application to regenerative medicine has been investigated in a wide variety of pathological conditions. In the case of skeletal bone tissues, the efficacy of MSC transplantation has been shown in applications to delayed unions and avascular necrosis. If iPSCs derived from BMSCs still possess the influence of their origins in early passages, they may differentiate into mesenchymal cells such as chondrocytes and osteoblasts more efficiently than iPSCs derived from dermal fibroblasts (DFs), which have little ability to differentiate into cells of other lineages. A simple comparison between established iPSCs derived from BMSCs and DFs may not answer this question, because the properties of iPSCs may be affected by the genomic information of each individual.

To overcome this, we have generated two types of iPSCs in this study, one derived from BMSCs (BM-iPSCs) and the other from DFs (DF-iPSCs) of the same donor, and compared gene expression profiles, DNA methylation status, and differentiation properties of each type. Although the capacity for differentiation differs among iPSC clones, there is no evidence that BM-iPSCs are superior to DF-iPSCs in terms of differentiation properties into the chondrogenic and osteogenic lineage, even though they are low-passage cells. Clone verification should be of critical importance to future regenerative medicine.

## Materials and Methods

### Preparation of BMSCs and DFs from the Same Donor

BMSCs were prepared from iliac bone as described previously [21] and expanded as a monolayer. Circular skin tissue was collected from the volar side of the forearm with a skin biopsy device (Kai medical, Gifu, Japan), cut into small pieces, and plated onto a plastic dish. After several days, fibroblastic cells appeared around the pieces and expanded. Informed consent was obtained from all donors with written consent, and these procedures were approved by the Ethics Committee of the Department of Medicine and Graduate School of Medicine, Kyoto University.

### Cell Culture

DFs and PLAT-A cells (kindly provided by Dr. T. Kitamura, University of Tokyo; [22]) were maintained in Dulbecco’s modified eagle medium (DMEM; Nacalai Tesque, Kyoto, Japan) containing 10% fetal bovine serum (FBS; Nichirei Inc., Tokyo, Japan) and 0.5% penicillin and streptomycin (Invitrogen Co., Carlsbad, CA). BMSCs were maintained in a minimal essential medium with GlutaMAX (Invitrogen) supplemented with 10% fetal bovine serum and 0.5% penicillin and streptomycin. iPSCs were generated and maintained in Primate ES cell medium (ReproCELL, Tokyo, Japan) supplemented with 4 ng/ml recombinant human basic fibroblast growth factor (bFGF; WAKO, Osaka, Japan).

### Establishment of iPSCs by Retroviral Infection

PLAT-A packaging cells were plated at 8×10^6^ cells per 100-mm type I collagen-coated dish (Corning Inc., Corning, NY) and incubated overnight. The next day, cells were separately transfected with pMXs vectors harboring four human Yamanaka factors (OCT3/4, SOX2, KLF4, and c-MYC) with FuGENE 6 transfection reagent (Roche, Basel, Switzerland). Twenty-four hours after transfection, the medium was collected as virus-containing supernatant. DFs and BMSCs were seeded at 1.5×10^5^ cells per 60-mm dish 1 day before infection. Before reseeding of DFs and BMSCs, 4 ng/ml of bFGF was added to the medium. Virus-containing supernatants were filtered through a 0.45-mm pore-size filter and supplemented with 4 mg/ml polybrene. Equal amounts of supernatant containing each of the four retroviruses were mixed, transferred to DFs and BMSCs, and incubated overnight. Six days after infection, DFs and BMSCs were harvested by trypsinization and replated at 1×10^5^ cells per 100-mm dish covered with mitomycin C-treated mouse embryonic fibroblasts cell line (SNL; [23]). The next day, the medium was replaced with Primate ES cell medium supplemented with 4 ng/ml bFGF. The medium was changed every other day. Thirty days after infection, colonies were picked up.

### RT-PCR and qPCR

Total RNA was purified with an RNeasy kit (Qiagen, Valencia, CA) and treated with a DNase-one kit (Qiagen) to remove genomic DNA. One microgram of total RNA was reverse transcribed for single-stranded cDNA using an oligo (dT) primer and Superscript III reverse transcriptase (Invitrogen), according to the manufacturer’s instructions. PCR was performed with ExTaq (Takara, Shiga, Japan). Quantitative PCR was performed with Power SYBR Green qPCR mastermix (Invitrogen) and analyzed with the 7300 real-time PCR system or StepOne real-time PCR system (Applied Biosystems, Forester City, CA). Human iPSCs (201B7) were used as a control [3]. Primer sequences are listed in Table S1. We repeated this assay at least two times and results showed a similar tendency.

### Karyotyping

The karyotype of each clone was analyzed by G-band staining using 50 metaphase cells (Nihon Gene Research Laboratories Inc., Sendai, Japan).

### Teratoma Formation

Feeder cells were removed by treatment with CTK solution containing 0.25% trypsin (Invitrogen), 0.1 mg/ml collagenase IV (Invitrogen), 0.1 mM CaCl_2_, and 20% KSR (Invitrogen). iPSCs were then harvested with scrapers, collected into tubes, centrifuged, and suspended in DMEM/F12 (Invitrogen). Half of the cells from a confluent 100-mm dish were injected subcutaneously into the dorsal flank of a SCID mouse (CREA, Tokyo, Japan). From eight to twelve weeks after injection, tumors were dissected and fixed with PBS containing 10% formaldehyde.

### Animal Welfare

Animal studies were carried out in strict accordance with recommendations in the Regulations on Animal Experimentation at Kyoto University. The protocol in this study was approved by the Animal Research Committee of Kyoto University. All injections were performed under anesthesia, and all efforts were made to minimize suffering. Mice were humanely sacrificed prior to tissue collection.

### Chondrogenic Differentiation

When the colonies reached 70–80% confluence, undifferentiated iPSCs on SNL feeder cells were used for differentiation. Cells were treated with CTK solution and rinsed twice with PBS to remove feeder cells. iPSCs were then collected with a scraper and suspended as clumps in EB formation medium (DMEM/10% KSR/10% FBS). EBs were cultured for 7 days on non-adherent petri dishes (bacterial petri dish, #31-001-002; IWAKI Co., Tokyo, Japan). The medium was changed every 3 days. Next, EBs were seeded onto 10-cm gelatin-coated dishes. After outgrowth cells became confluent (within 10 days), cells were incubated with 0.25% trypsin/EDTA at 37°C for 3–5 min, filtrated through a cell strainer (70 µm, #352350; BD falcon, Franklin Lakes, NJ), and re-seeded on new gelatin-coated dishes. Once confluent (5–7 days), cells were collected with trypsin/EDTA. Cells (2.5×10^5^) were placed in a 15-ml polypropylene tube, centrifuged at 1200 rpm for 3 min at room temperature, and re-suspended in chondrogenic medium (hMSC Chondrogenic Differentiation BulletKit®; Lonza, Basel, Switzerland) supplemented with 10 ng/ml TGFβ3 (R&D system, Minneapolis, MN). Cells were re-centrifuged and maintained as a small pellet for 14 days. The culture medium was replaced every three days.

### Glycosaminoglycan (GAG) Value

GAG content in pellets was quantified with BLYSCAN Dye and Dissociation reagents (BIOCOLOR, Belfast, UK). DNA content was quantified using a PicoGreen dsDNA Quantitation kit (Invitrogen).

### Histology

Pellets were fixed with PBS containing 4% paraformaldehyde overnight after 14 days of induction. Fixed pellets were dehydrated with a series of graded alcohol solutions, cleaned by treatment with Clear Plus (FALMA, Tokyo, Japan), and infiltrated with paraffin. Paraffin-embedded sections were rehydrated and stained with Alcian blue and eosin or with hematoxylin and eosin (WAKO).

### Osteogenic Differentiation

For osteogenic induction from iPSCs, EBs were formed as shown above. During EB formation and osteogenic induction, 100 ng/ml Activin-A was added to each medium. EBs were seeded on a 10-cm gelatin-coated dish. After 5 days, outgrowth cells were collected and filtered through a cell strainer. A total of 2×10^6^ cells/well were seeded on a 6-well gelatin-coated dish and cultured in osteogenic induction medium (αMEM, 10% FBS, 0.1 µM Dexamethasone, 50 µg/ml Ascorbic acid, 10 mM β-glycerophosphate). The culture medium was replaced every three days.

### DNA Microarray

Total RNA was prepared using the RNeasy Mini Kit (Qiagen). cDNA was synthesized using the GeneChip WT (Whole Transcript) Sense Target Labeling and Control Reagents kit as described by the manufacturer (Affymetrix, Santa Clara, CA, USA). Hybridization to GeneChip Human Gene 1.0 ST expression arrays, washing, and scanning were performed according to the manufacturer’s protocol (Affymetrix). Data were analyzed using GeneSpring GX 11.5.1 (Agilent Technologies) for scatter plotting, hierarchical clustering, and Venn diagram generation. For hierarchical clustering, we used the Chebyshev correlation for similarity measures and for average linkage clustering. Reported microarray data have been deposited in the public database Gene Expression Omnibus (http://www.ncbi.nlm.nih.gov/geo/) under accession no. GSE41202.

### Genome-wide DNA Methylation Profiling

To analyze the genome-wide DNA methylation status, we performed Illumina’s 450 K Infinium methylation assay according to the manufacturer’s protocol. The 450 K data was subtracted from the background and normalized to the control in the GenomeStudio. For clustering analysis, we first selected probes of differentially methylated regions (DMR) between DFs and BMs (difference of average beta value, >0.5). Hierarchical clustering was performed with hclust program in R, with Euclidian distance and average linkage using additional ES data in GSE31848.

## Results

### Generation and Characterization of Human iPSCs from BMSCs and DFs of the Same Donor

**Figure 1:**
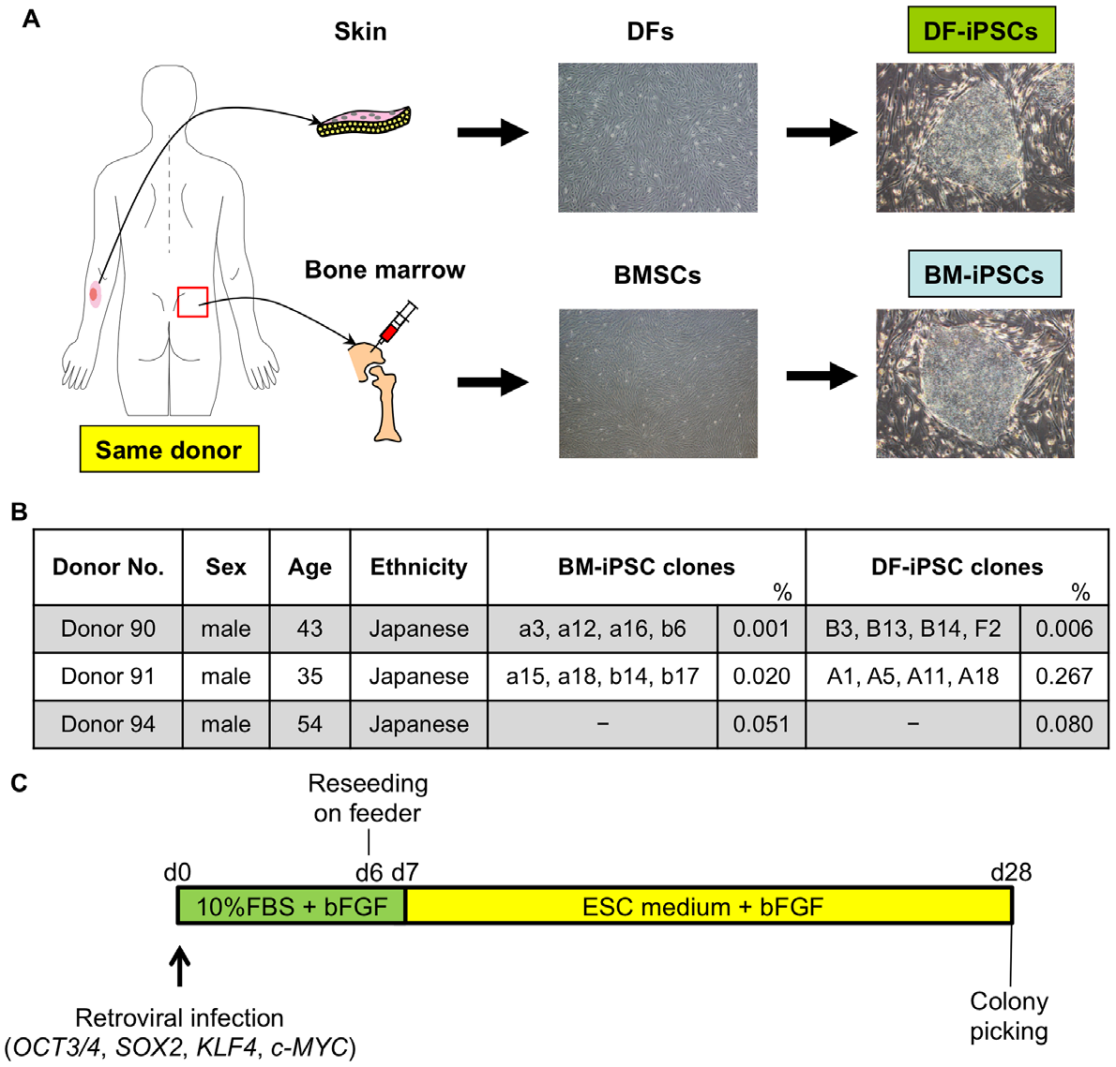
Generation of iPSCs from BMSCs and DFs of the same donor. A) Schematic representation of the generation of iPSCs from dermal fibroblasts (DFs) and bone marrow stromal cells (BMSCs) of the same healthy donor. B) Donor information and established clones’ names. %, iPSC derivation efficiency. -, not established as validated clones. C) Time course of iPSC generation.

**Figure 2:**
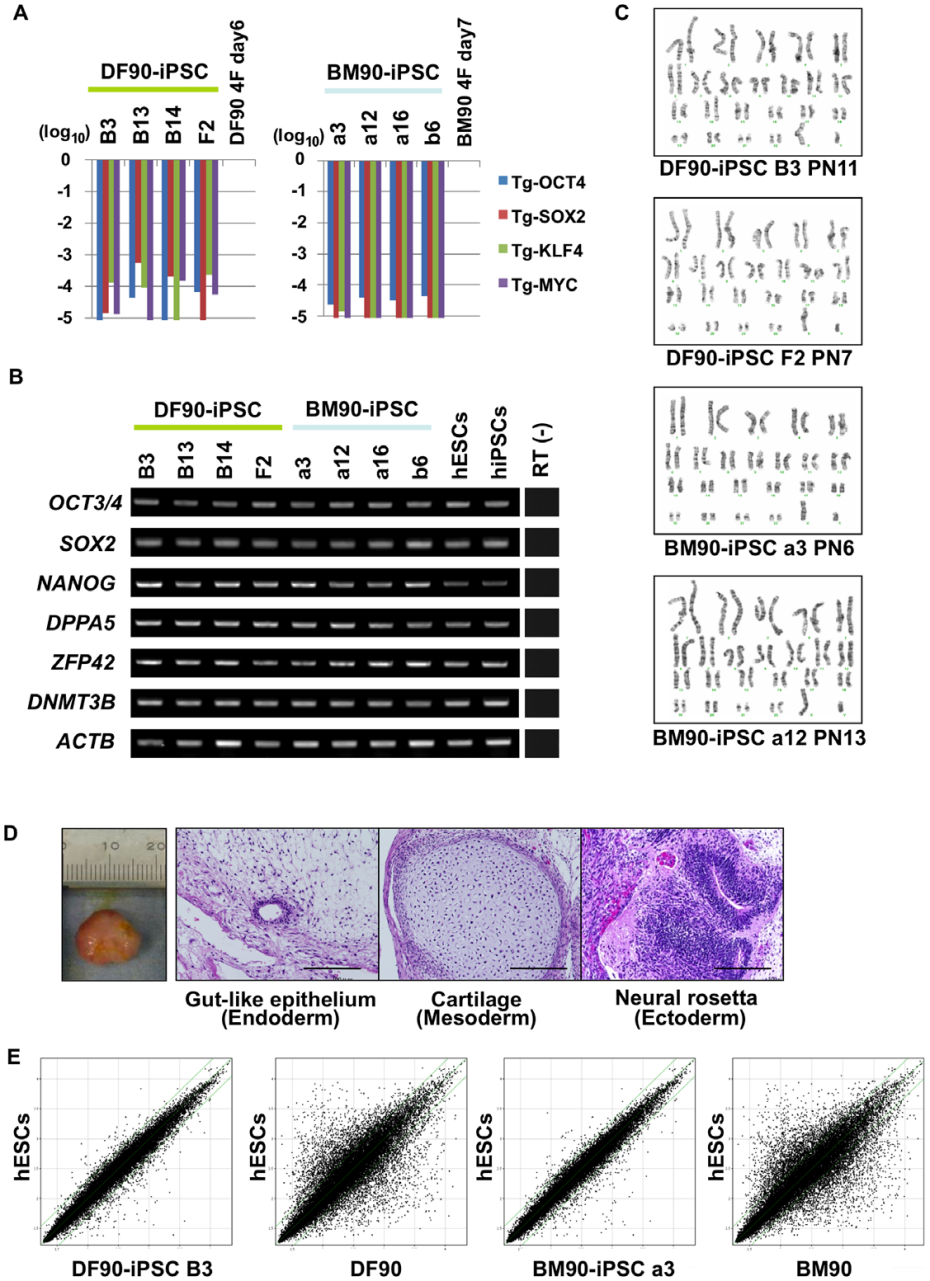
Characterization of iPSCs. A) Relative expression of retroviral transgenes in DF90 (left panel) and BM90 (right panel)-iPSC clones analyzed by RT-qPCR. The value of each transgene 6 days after infection of DF90 (DF90 4F day 6) and 7 days after infection of BM90 (BM90 4F day 7) was set to 1, and relative values of four transgenes in each clone are shown on a log scale. We selected four clones of which transgene expression was silenced less than 1/1000 compared to the control. B) Expression of ESC-marker genes. Primers used for OCT3/4 and SOX2 detect transcripts from endogenous genes, but not retroviral transgenes. All clones express ESC-marker genes similar to hESCs (KhES3) and hiPSCs (201B7). C) Karyotype analyses. PN, passage number. D) Histological analyses of teratomas derived from iPSCs (DF90-iPSC B3) by hematoxylin and eosin staining. Typical tissue features of each of the three germ layers were found. Scale bar, 200 µm. E) Comparison of global gene-expression patterns between each iPSC clone and hESCs (H9). The two green lines above and below the diagonal green lines indicate the boundary of 2-fold changes between the two samples.

We isolated paired BMSCs and DFs from the same healthy donor in three cases, with ages ranging from 35 to 54 (Figures 1A, B). Paired BMSCs and DFs from each donor were designated BM90 and DF90, BM91 and DF91, and BM94 and DF94. OCT3/4, SOX2, KLF4, and c-MYC were introduced with retroviral infection into each cell group at passage 1–2 (BMSCs) and passage 5–7 (DFs) (Figure 1C). ESC-like colonies appeared at day 28 after infection. The colony forming efficiency in DF91 was comparable to that reported previously, but that in BM91 was less than in DF91 (DF91, 0.267%; BM91, 0.020%; Figures 1B and S1). Similar results were obtained in the case of donor 90 and 94 (DF90, 0.006%; BM90, 0.001%; DF94, 0.080%; BM94, 0.051%; Figure 1B). Twenty nine, twenty one, eleven, forty seven, twenty, and thirteen colonies of DF90, BM90, DF91, BM91, DF94, and BM94, respectively, were randomly picked up and expanded as clones. To select clones for further investigation, we primarily evaluated the grade of silencing of exogenous OCT3/4, and then checked silencing of other transgenes (SOX2, KLF4, and c-MYC), karyotype abnormalities, and the expression of pluripotent stem cell markers (Figures 2A, B, C, S2, and S3). As a result, four clones of DF90-iPSCs (B3, B13, B14, and F2), four clones of BM90-iPSCs (a3, a12, a16 and b6), four clones of DF91-iPSCs (A1, A5, A11 and A18), and four clones of BM91-iPSCs (a15, a18, b14 and b17) were selected (Figure 1B; BM94 and DF94 were used for estimations of reprogramming efficiency and transgene silencing, but not for further analyses). All clones tested developed teratomas in vivo, which contained tissues featuring each of the three germ layers (DF90-iPSC B3, B13, B14, F2, BM90-iPSC a3, a12, b6, DF91-iPSC A1, A18; Figures 2D and S4). Global gene expression profiles of DF90- or BM90-iPSC clones were comparable to those of human ESCs (Figures 2E, S5; correlation coefficients between each iPSC clone >0.985). Similar results were obtained in DF91- and BM91-iPSC clones (Figure S5). All these data indicate that the selected clones satisfy criteria for iPSCs [24], [25].

### BM- and DF-iPSCs were Indistinguishable by Global Gene Expression Profiles

**Figure 3:**
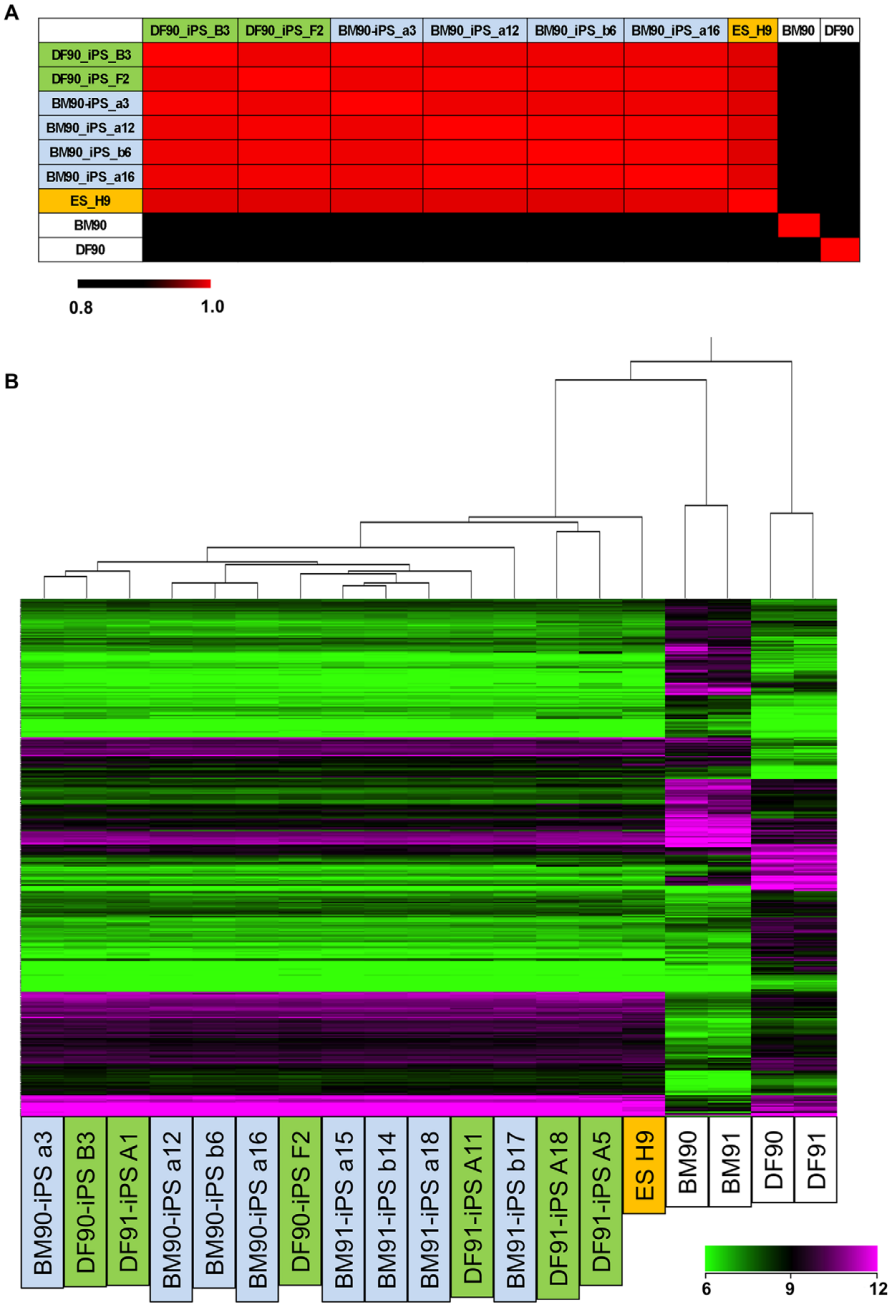
Global gene expression profiles do not differ between BM- and DF-derived iPSCs. A) Correlation coefficients between each cell were calculated using gene sets differentially expressed in DFs and BMSCs. Differentially expressed genes were defined as genes of which expression varied 2-fold in all pairs, between each BMs and each DFs (9 pairs). Passage number (PN) of cells were as below; BM90 (PN 1), BM 91 (PN 3), BM 94 (PN 2), DF90 (PN 5), DF 91 (PN 5), DF 94 (PN 5), DF90-iPSC B3 (PN 9), F2 (PN 4), BM90-iPSC a3 (PN 4), a12 (PN 4), a16 (PN 4), b6 (PN 4), DF91-iPSC A1 (PN 5), A5 (PN 6), A11 (PN 5), A18 (PN 7), BM91-iPSC a15 (PN 4), a18 (PN 4), b14 (PN 4), b17 (PN 4). B) Hierarchical clustering analysis in iPSC clones and hESC lines (H9) using differentially expressed gene sets in DFs and BMSCs.

**Figure 4:**
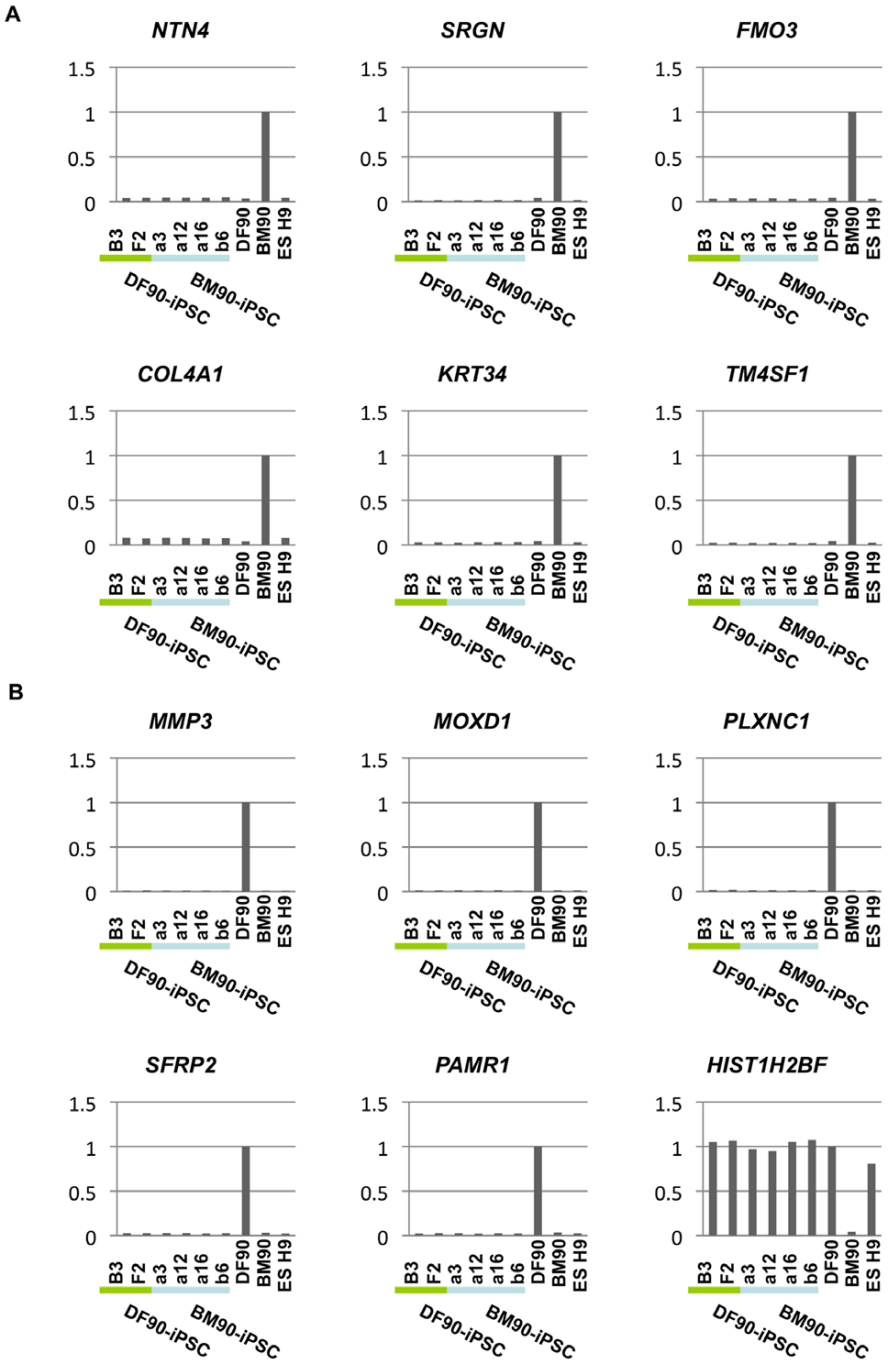
Reprogramming extinguishes lineage-specific gene expression. We presented the top six genes that were BMSC-specific (A) or DF-specific (B). Expression in BM90 (A) or DF90 (B) was set to 1 in each graph.

We next compared gene expression patterns of each iPSC clone since it was reported that low-passage human iPSCs retained a transcriptional memory of the original cells [26]. First, we compared global gene expression profiles of BM90, 91, and 94 with those of DF90, 91, and 94, and found that 356 genes were commonly up- (171 genes) or down- (185 genes) regulated in BMSCs (over two fold; Table S2). These genes, however, were not differentially expressed between DF90- and BM90-iPSC clones or DF91- and BM91-iPSC clones. Correlation coefficients were over 0.986 (donor 90 clones, Figure 3A) and 0.976 (donor 91 clones, Figure S6). Hierarchical clustering analysis in the donor 90 and 91 clones using the differentially expressed gene sets produced mixed branches regardless of their origins (Figure 3B). Most of the differentially expressed genes were silenced in BM- and DF-iPSC clones in a similar fashion (Figures 4A, B). These data indicated that BM- and DF-iPSC clones did not possess gene expression profiles of the original cells, and that BM- and DF-iPSCs were indistinguishable by gene expression.

### DNA Methylation Profiles of BM- and DF-iPSCs were General, but had Distinct Features

**Figure 5:**
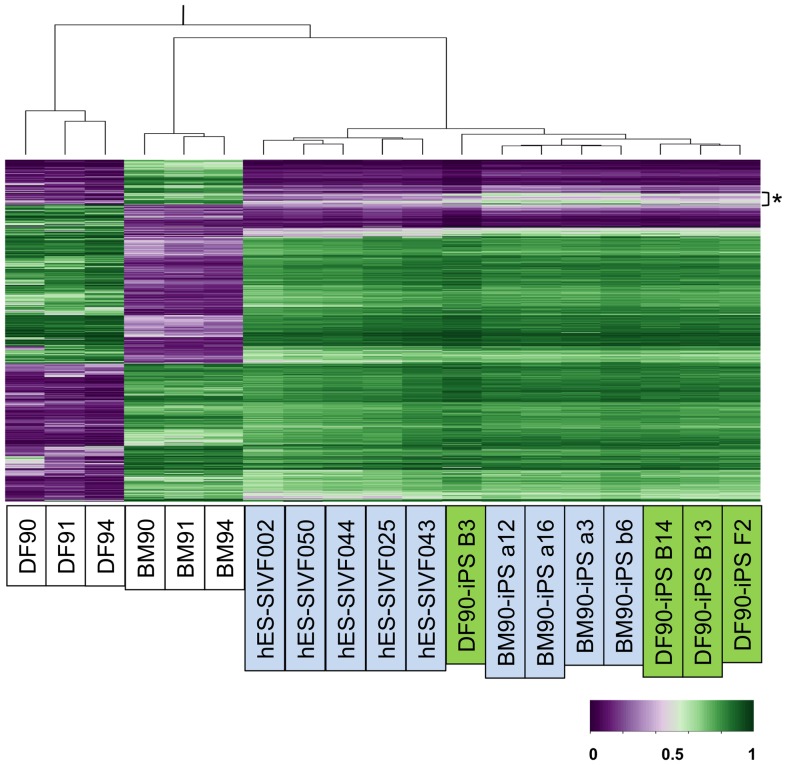
DNA methylation profiles of BM- and DF-iPSCs were similar in general, but had distinct features. Hierarchical clustering using Euclidian distance with probe sets of differentially methylated regions between DFs and BMSCs.

Since most published studies identified epigenetic differences among iPSCs from different origins [10], [11], [14], [15], [27], [28], [29], we next analyzed genome-wide DNA methylation profiles of DFs, BMSCs, and each iPSC clone. Using probe sets of differentially methylated regions (DMR) between DFs and BMSCs (6,176 in 485,531 probes), the overall methylation patterns of iPSC clones were similar to each other and those of ESCs, and distinct from those of original DFs and BMSCs (Figure 5). Hierarchical clustering and heat map analyses, however, revealed a set of DMR probes shared by iPSC clones derived from one origin but not from the other. These observations indicate that a certain level of tissue memory is preserved in iPSCs derived from DFs and BMSCs. To gain insight into the character of each iPSC clone, we focused on loci near the top 30 DF-specific and BM-specific genes (15 in 185 genes and 15 in 171 genes, respectively), and also genes important for chondrogenic and osteogenic differentiation (nine and nine genes, respectively). The methylation status of these loci changed with reprogramming processes and differences were almost diminished in all iPSC clones regardless of their origins (data not shown).

Taken together, these results revealed that iPSC clones derived from BMs and DFs are similar in global DNA methylation status, whereas they retain epigenetic memory during the reprogramming process.

### Cell-autonomous Differentiation Properties of iPSCs Differ with Clones Regardless of their Developmental Origin

**Figure 6:**
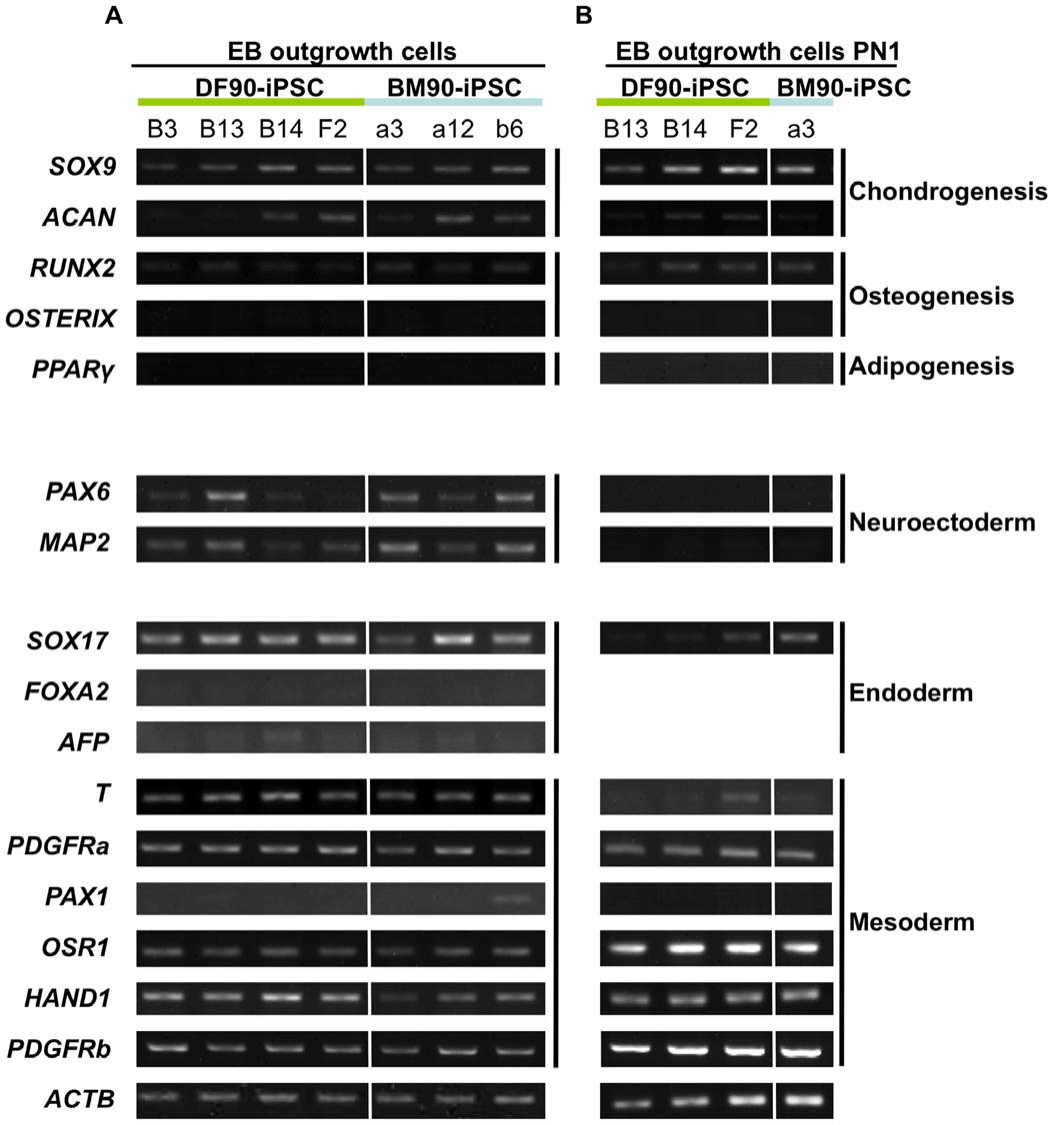
The propensity for EB-mediated cell-autonomous differentiation in iPSC clones differs regardless of developmental origin. Gene expression of EB outgrowth cells (A) and reseeded cells (PN1; B). RNA was extracted from cells that reached semi-confluency. RT-PCR was performed with genes related to chondrogenesis (SOX9 and ACAN), osteogenesis (RUNX2 and OSTERIX), or adipogenesis (PPARγ). The capacity to differentiate into bone and cartilage lineages was similar between DF- and BM-iPSC clones. The expression of genes representative of each of the three germ layers was also analyzed, which showed no difference among clones.

Several reports demonstrated that low-passage iPSCs exhibited the differentiation properties of original cell types [11], [14], [15], [27], [28], [30], [31], [32], while others contradicted it [10], [29], [33], [34]. To investigate the existence of such intrinsic differentiation properties of our iPSC clones, EBs were formed using low passage DF90- and BM90-iPSCs (between passage 12–20), and then transferred into a monolayer culture system. RNAs were extracted from cells outgrowing from EBs. Each clone had its own tendency to express markers for ectodermal, mesodermal, and endodermal lineages, and there were no data to support BM-iPSC clones’ tendency to differentiate into bone and cartilage lineages (Figure 6A). Similar results were obtained when we analyzed cells passaged once (Figure 6B). Experiments using another pair gave similar results (Figure S7A). Based on these results, we conclude that clonal differences, rather than original cell types, affect the differentiation property of human iPSCs even if they are of low-passage-number.

### Induced Differentiation Properties of iPSCs Differ with Clones, but not with Original Cell Types

Finally, differentiation properties after induction were compared between DF- and BM-iPSC clones. Chondrogenic and osteogenic differentiation was used since BMSCs are more potently differentiated into chondrocytes, osteoblasts, and adipocytes than DFs (Figure S8).

**Figure 7:**
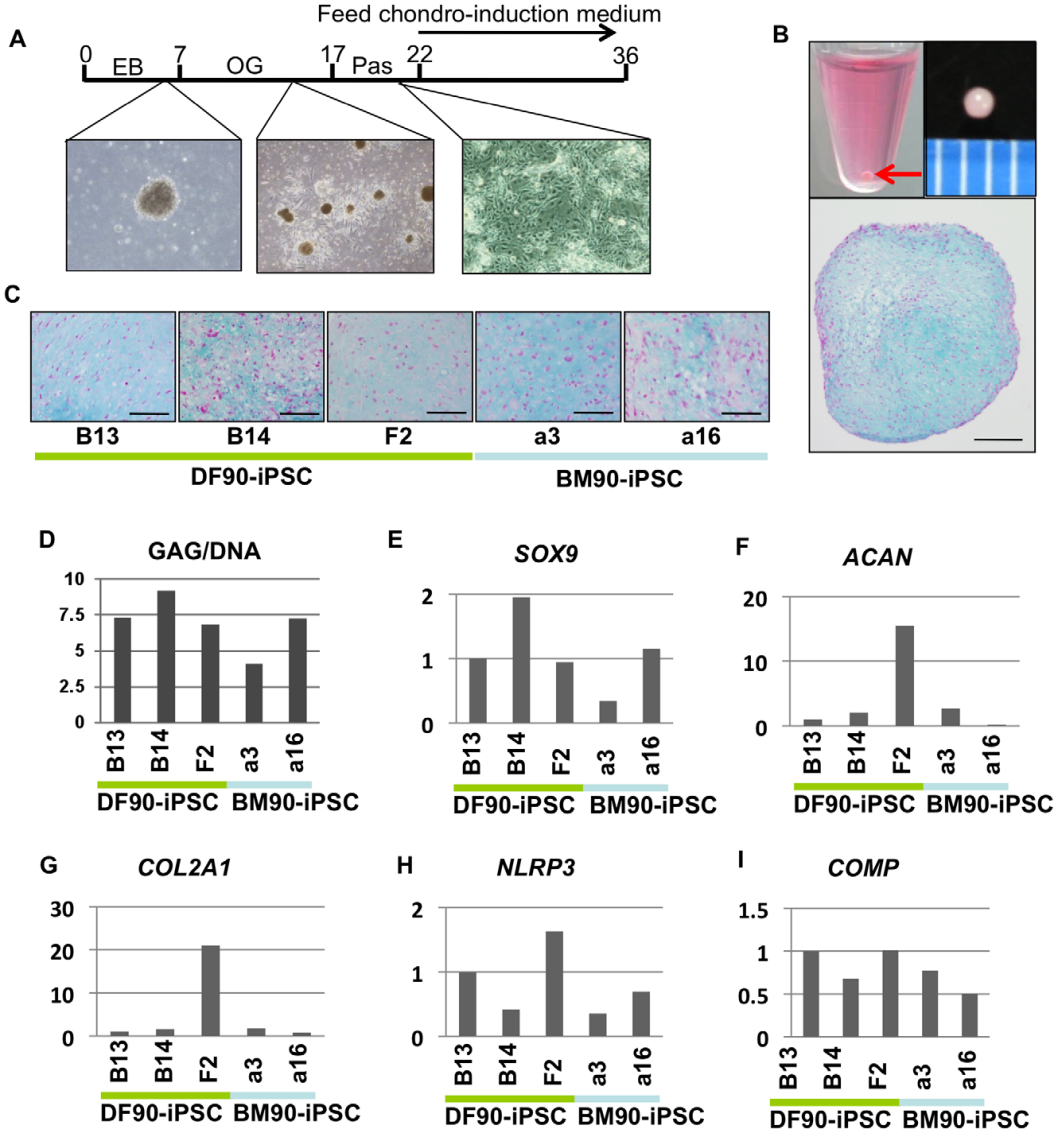
Induction of chondrogenic differentiation in iPSCs. A) Time course of chondrogenic differentiation. EB, embryoid body. OG, outgrowth. Pas, passaged once. B) Macroscopic views and Alcian blue staining of a section of a pellet. The red arrow indicates a pellet at the bottom of a 15-ml conical tube. Middle column, scale bar, 1 mm. Right column, scale bar, 200 µm. C) Alcian blue staining of sections of pellets. Scale bar, 100 µm. D) GAG/DNA of pellets. GAG/DNA differed with clones regardless of cell-of-origin. E-I) Comparison of the relative expression of chondrogenesis-related genes (SOX9, COL2, ACAN NLRP3 and COMP) by RT-qPCR. The value of DF-iPSC B13 was set to 1 in each experiment.

Cells outgrowing from EBs were expanded, passaged once, and then used for the 3D pellet culture system (Figures 7A, B). These procedures enriched mesodermal and endodermal cells and omitted neuronal cells (compare Figure 6A with 6B). After fourteen days of induction, we observed extracellular matrix-rich chondrocyte-like cells in the DF90 and BM-90 iPSC clones analyzed (Figure 7C). GAG content was increased in all clones at comparable but various levels (Figures 7D and S7B), and the expression of SOX9, a master transcriptional regulator of chondrogenesis, was induced in accordance with GAG content (Figure 7E). We checked the expression of cartilage-specific genes, Aggrecan (ACAN), COL2A1, NLRP3, and COMP, and found that clone F2 in DF-iPSCs expressed these genes at higher levels than other clones (Figures 7F–I).

**Figure 8:**
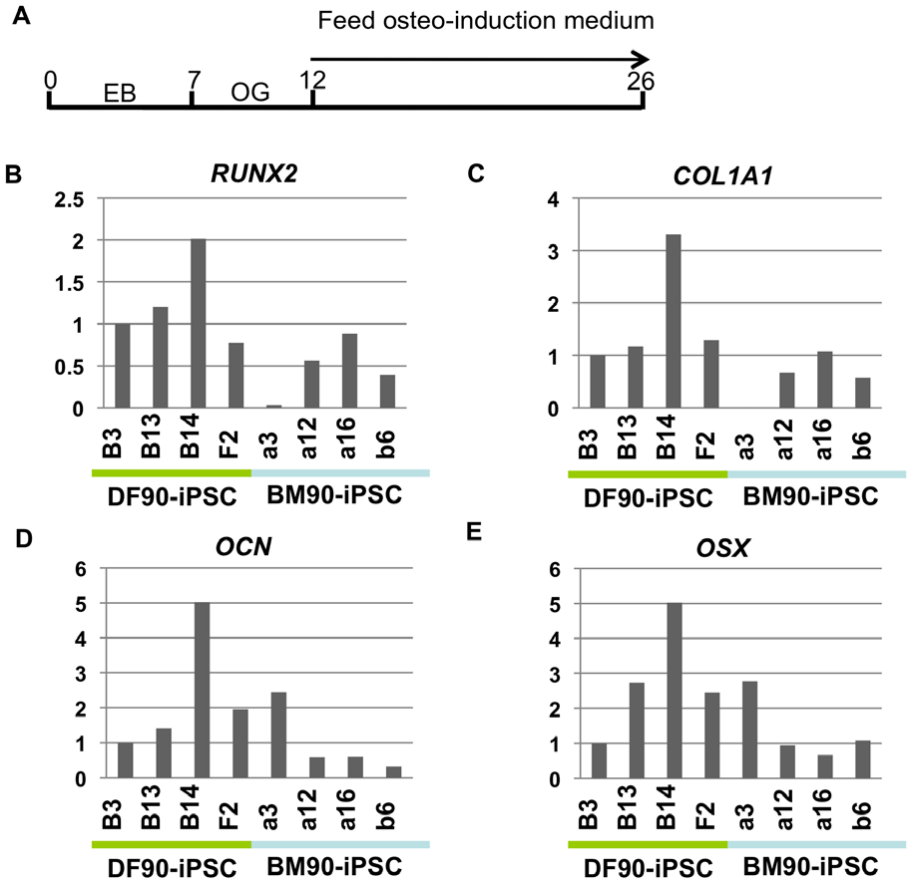
Induction of osteogenic differentiation in iPSCs. A) Time course of osteogenic differentiation. EB, embryoid body. OG, outgrowth. B-E) Comparison of the relative expression of osteogenesis-related genes (RUNX2, COL1A1, OCN and OSX) by RT-qPCR. The value of DF-iPSC B13 was set to 1 in each experiment.

For osteogenic induction, we used cells outgrowing from EBs, plated them at a high density, and cultured them for fourteen days in medium commonly used for the osteogenic induction of MSCs (Figure 8A). The expression of osteogenic markers was induced higher in DF-derived clone B14 than other clones (Figures 8B–E). Statistical analyses revealed that, although we recognized a tendency that DF-derived iPSCs are well differentiated into osteogenic and chondrogenic lineages compared with BM-derived iPSCs, there are no significant differences between iPSCs from BMs and DFs (Figure S9). Taken together, these results indicate that BM-iPSCs are not superior to DF-iPSCs in terms of differentiation properties into the chondrogenic and osteogenic lineage.

## Discussion

We generated human iPSCs derived from BMSCs and DFs of the same donor, and compared differentiation properties of genetically matched iPSC clones with different origins. We observed no marked differences between BM- and DF-iPSCs in global gene expression profiles. BM- and DF-iPSCs also share a general methylation profile but have a set of methylation features related to cell-of-origin. Differentiation properties for chondrogenic and osteogenic lineages by our induction methods, however, showed no clear difference between BM- and DF-iPSCs. These data in part contradict the prevailing theory that tissue origins affect the differentiation potentials of iPSC both in mice [14], [27] and humans [11], [15]. However, at least two groups reported that regardless of their epigenetic memory, human iPSCs derived from distinct origins could efficiently differentiate into an endoderm or hepatic lineage [10]. Therefore, the relationship between tissue memory and differentiation properties is still controversial. Our results shown here indicate that the differentiation property of iPSCs into at least two types of mesenchymal cells (chondrocyte and osteoblast) differs with clones, but not the cell type of origin. Different results may be obtained in different types of mesenchymal cells or by different methods for differentiation.

We observed clonal differences regardless of cell type of origin, consistent with several other reports [10], [29], [33], [34]. It has been reported that epigenetic and transcriptional properties of iPSC clones affect their capacity to differentiate into several lineages. Our transcriptome analyses showed no correlation between global gene expression profiles and differentiation capacities (Figures 3 and 4). When we hypothesized representative iPSCs by averaging the gene expression of iPSC clones of the same origin and donor, hierarchical clustering separated iPSCs according to donors (Figure S10), suggesting the existence of donors’ memory and the importance of comparing iPSC clones derived from the same donor. Recently, a bioinformatic scorecard was proposed to predict the differentiation efficiency of individual human ESC lines and iPSC clones [35], [36]. Although they used iPSC clones derived from varying characteristics including age, sex, and health status, comparisons of scorecards drawn by genetically matched iPSC clones may be a future challenge.

This is the first report that compares chondrogenic properties between iPSC clones. Using our induction method, we found that expression of chondrogenic markers varied with clones regardless of cell type of origin (Figures 7E–I). We repeated this assay at least two times and results showed a similar tendency (data not shown). Although we detected expression of chondrogenic lineage markers after induction, immunohistochemical analysis revealed that the number of type II collagen-positive cells was very small (data not shown). To compare the number of chondrocytes induced by a different method, we generated teratomas, made serial sections, and calculated the size of Alcian blue-positive chondrogenic areas (Figure S11). Although preliminary analysis showed no preference for chondrogenesis between DF- and BM-iPSC clones, it was difficult to perform quantitative analyses because the chondrogenic area varied with sections and induction efficiency was too low (around 1% in sections, ranging from 0% to 1.6%; Figure S11). Moreover, clone F2, which exhibited a relatively high chondrogenic capacity through our 3D pellet culture method, had minimum chondrogenic areas in teratomas. Differences in chondrogenic efficiency depending on the methods may have reflected some clonal characteristics (i.e. different responses to exogenous factors), which we will address in the near future.

We used BMSCs as a source of cells to be reprogrammed because our primary working hypothesis was that BMSCs, including mesenchymal stem cells (MSCs), exhibit higher efficiency of reprogramming and/or require fewer Yamanaka factors such as neural stem cells (NSCs) [37]. Our results, however, showed less efficiency of reprogramming in BMSCs (Figure S1). We also failed to generate iPSCs from BMSCs by infecting 3 factors (without c-MYC; data not shown). In contrast to our observations, one report showed that mouse MSCs purified with FACS exerted a higher reprogramming rate and produced high-quality iPSCs [38]. That report also found that transgenes were completely silenced in iPSC clones derived from MSCs. However, we observed no differences in efficiency of transgene silencing between BM- and DF-iPSC clones (Figure S12). Although continuous expression of transgenes has already been overcome using episomal vectors or other excisable gene delivery systems [39], it is still intriguing how transgenes are efficiently silenced.

## Supporting Information

Figure S1 **Ratio of ALP-positive colonies and transduction efficiency of retrovirus in DFs and BMSCs.** Upper panel shows the ratio of ALP-positive colonies per plated DFs or BMs (1×10^5^ cells). Lower panel shows fluorescence micrographs indicating transfection efficiency. Shown are percentages of cells expressing GFP.
(TIF)

Figure S2 **Expression levels of transgenes and ESC-marker genes of each DF91- and BM91-iPSC clone. hESCs, KhES3, hiPSCs, 201B7.**
(TIF)

Figure S3 **Karyotypes of each iPSC clone.**
(TIF)

Figure S4 **Teratomas derived from each iPSC clone.** Hematoxylin and eosin staining of teratomas derived from each iPSC clone showed differentiation in three germ layers.
(TIF)

Figure S5 **Global gene expression patterns compared between each iPSC clone and hESCs.** Global gene expression patterns were compared between each iPSC clone and hESCs (H9) with microarrays. The two green lines above and below the diagonal green lines indicate the boundary of 2-fold changes between the two samples.
(TIF)

Figure S6 **Correlation coefficients between each cell from donor 91 were calculated using gene sets differentially expressed in DFs and BMSCs.**
(TIF)

Figure S7 **The propensity for differentiation in iPSC clones derived from donor 91 differs regardless of developmental origin.** A) The propensity for EB-mediated cell-autonomous differentiation in iPSC clones (donor 91) differs regardless of the developmental origin. B) Induction of chondrogenic differentiation in iPSCs (donor 91). GAG/DNA differed with clones regardless of cell-of-origin.
(TIF)

Figure S8 **Chondrogenic, osteogenic, and adipogenic differentiation assays with the original BMSCs and DFs.** A) Macroscopic views and Alcian blue staining of a section of a pellet (left panel) and expression of chondrogenesis-related genes (SOX9 and COL2) by RT-PCR (right panel). B) Alizarin red staining of osteogenic induction samples (left panel) and calcium contents (right panel). C) Oil-Red-O staining (left panel) and the amount of triglycerides (TG). We used DFs at passage 5–7 and BMs at passage 1–2 for differentiation and confirmed that the DFs used in this study could not differentiate into either chondrocytes, osteoblasts, or adipocytes. Experiments were performed as described previously [21].
(TIF)

Figure S9 **Statistical analyses of differentiation potentials between DF-derived and BM-derived iPSCs.** A) Chondrogenic markers. B) Osteogenic markers. Each dot corresponds to each clone. *P* values are 0.36 (SOX9), 0.49 (ACAN), 0.49 (COL2A1), 0.37 (NLRP3), 0.23 (COMP), 0.052 (RUNX2), 0.11 (COL1A1), 0.24 (OCN), and 0.19 (OSX) (Unpaired *t* tests). n.s., not significant.
(TIF)

Figure S10 **Hierarchical clustering analysis of iPSCs.** BM90-iPSCs (average of BM90-iPSC a3, a12, a16, and b6), DF90-iPSCs (average of DF90-iPSC B3 and F2), BM91-iPSCs (average of BM91-iPSC a15, a18, b14, and b17), DF91-iPSCs (average of DF91-iPSC A1, A5, A11, and A18), and hESCs (H9) were subjected to clustering analysis using all gene sets.
(TIF)

Figure S11 **Ratio of cartilage area in teratomas.** The cartilage area in teratomas was investigated. Five sections were prepared. Total area and cartilage area detected by Alcian blue staining were calculated using software in BIOREVO (Keyence, Osaka, Japan).
(TIF)

Figure S12 **Ratio of transgene-silenced clones.** The ratio of clones in which retroviral transgene expression was silenced was less than 1/1000 compared to controls (the value of each transgene 6 days after infection of DF (DF 4F day 6) and 7 days after infection of BM (BM 4F day 7)).
(TIF)

Table S1 **Primer sequences.**
(XLS)

Table S2 **Genes differentially expressed in DFs and BMSCs.** A) Genes highly expressed in DFs compared with BMSCs. B) Genes highly expressed in BMSCs compared with DFs.
(XLS)
