# Supplementary figures and images for: Genetically Matched Human iPS Cells Reveal that Propensity for Cartilage and Bone Differentiation Differs with Clones, not Cell Type of Origin
Source: PLoS One. 2013 Jan 31;8(1):e53771. doi: 10.1371/journal.pone.0053771 (PMC3561398; doi:10.1371/journal.pone.0053771)

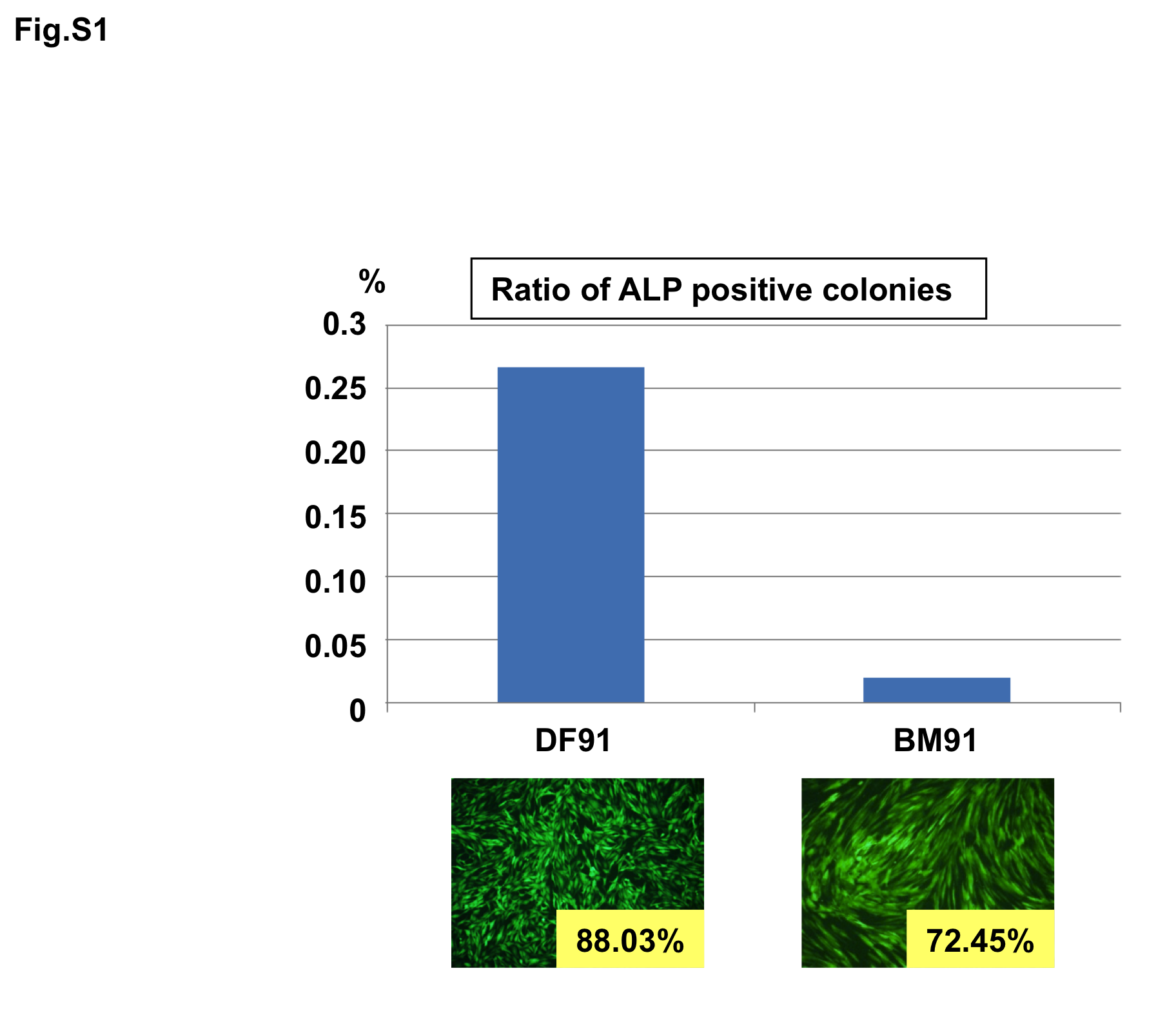

Supplement: Figure S1 — Ratio of ALP-positive colonies and transduction efficiency of retrovirus in DFs and BMSCs. Upper panel shows the ratio of ALP-positive colonies per plated DFs or BMs (1×10^5^ cells). Lower panel shows fluorescence micrographs indicating transfection efficiency. Shown are percentages of cells expressing GFP. (TIF) [file pone.0053771.s001.tif]

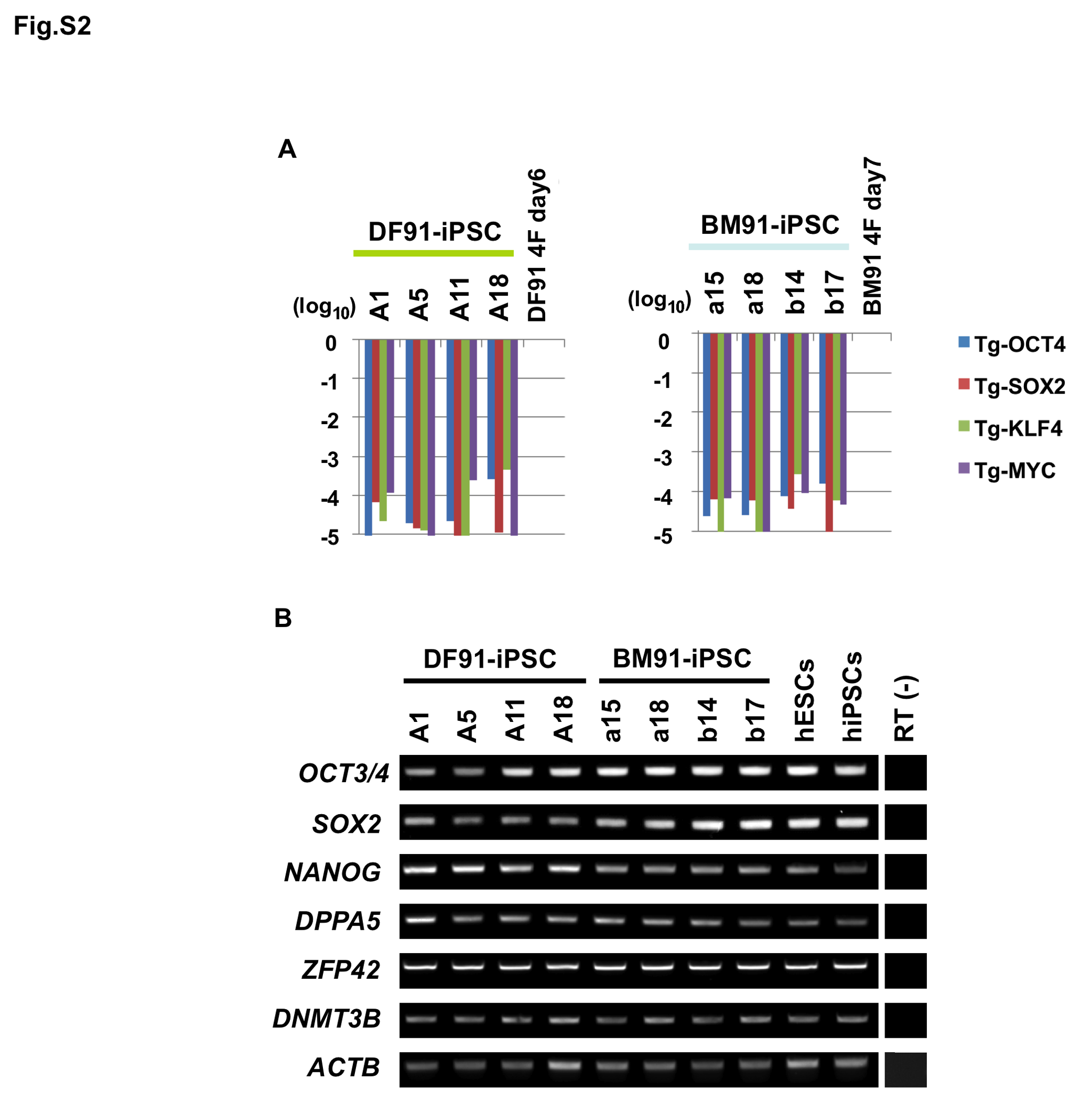

Supplement: Figure S2 — Expression levels of transgenes and ESC-marker genes of each DF91- and BM91-iPSC clone. hESCs, KhES3, hiPSCs, 201B7. (TIF) [file pone.0053771.s002.tif]

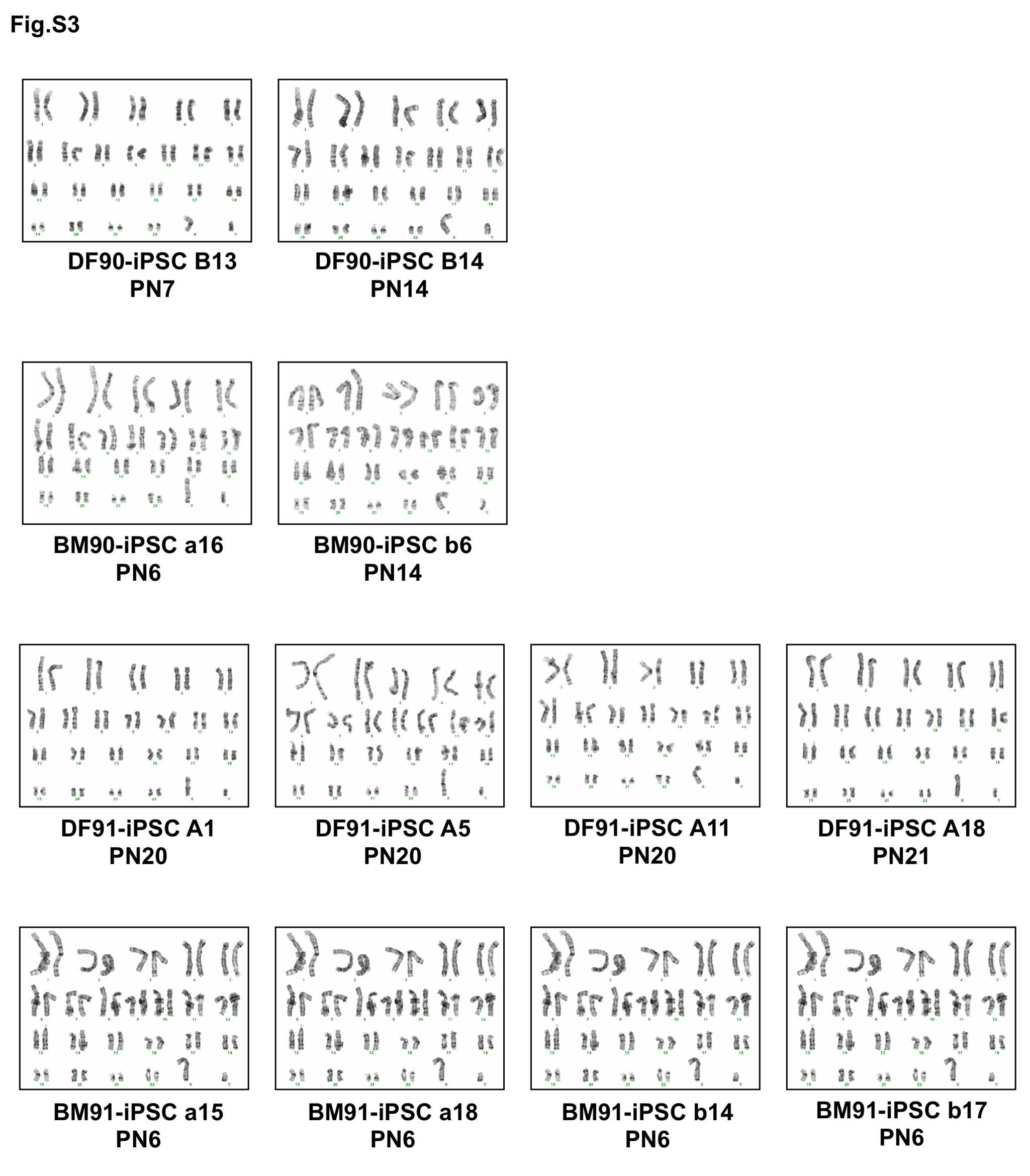

Supplement: Figure S3 — Karyotypes of each iPSC clone. (TIF) [file pone.0053771.s003.tif]

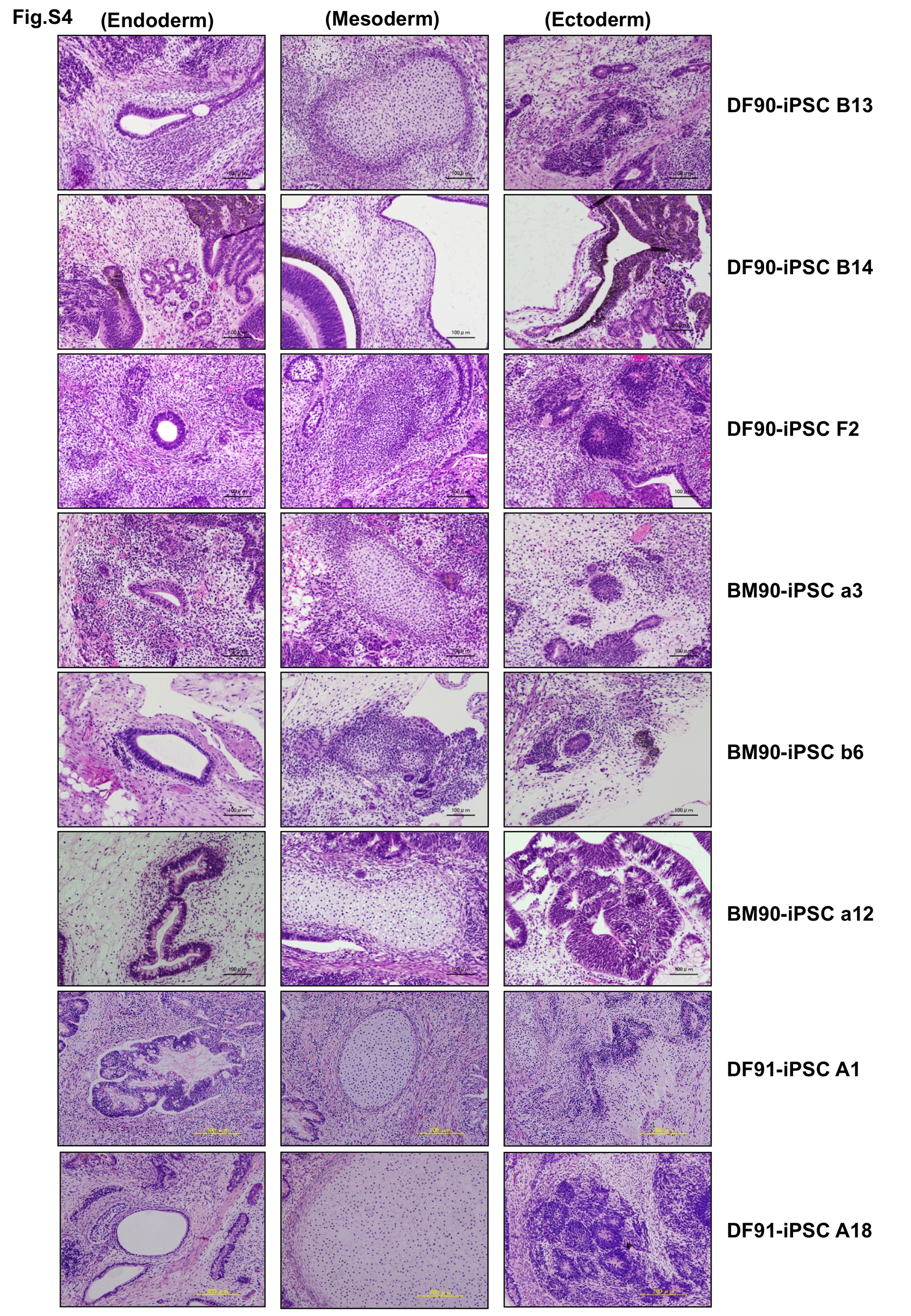

Supplement: Figure S4 — Teratomas derived from each iPSC clone. Hematoxylin and eosin staining of teratomas derived from each iPSC clone showed differentiation in three germ layers. (TIF) [file pone.0053771.s004.tif]

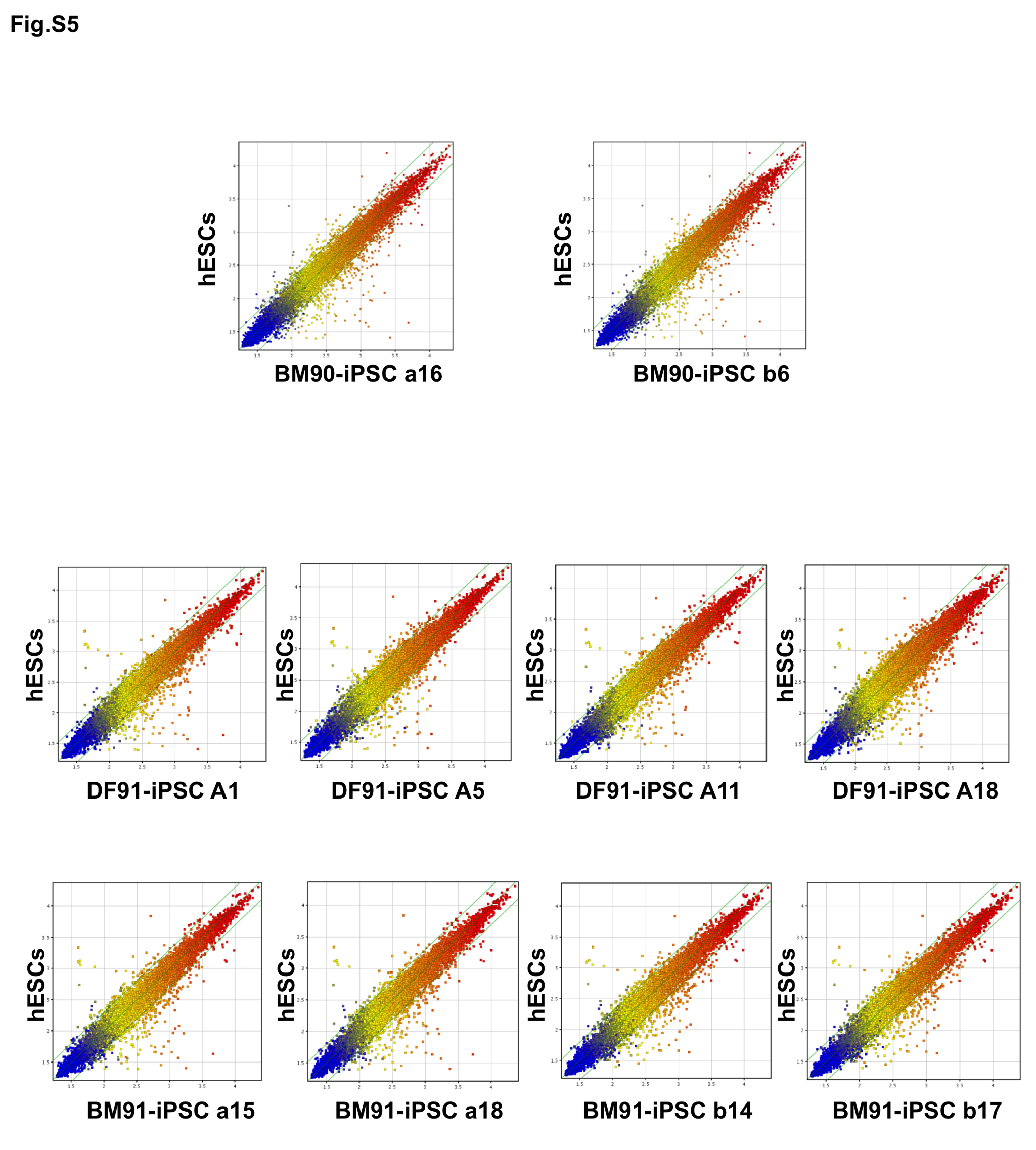

Supplement: Figure S5 — Global gene expression patterns compared between each iPSC clone and hESCs. Global gene expression patterns were compared between each iPSC clone and hESCs (H9) with microarrays. The two green lines above and below the diagonal green lines indicate the boundary of 2-fold changes between the two samples. (TIF) [file pone.0053771.s005.tif]

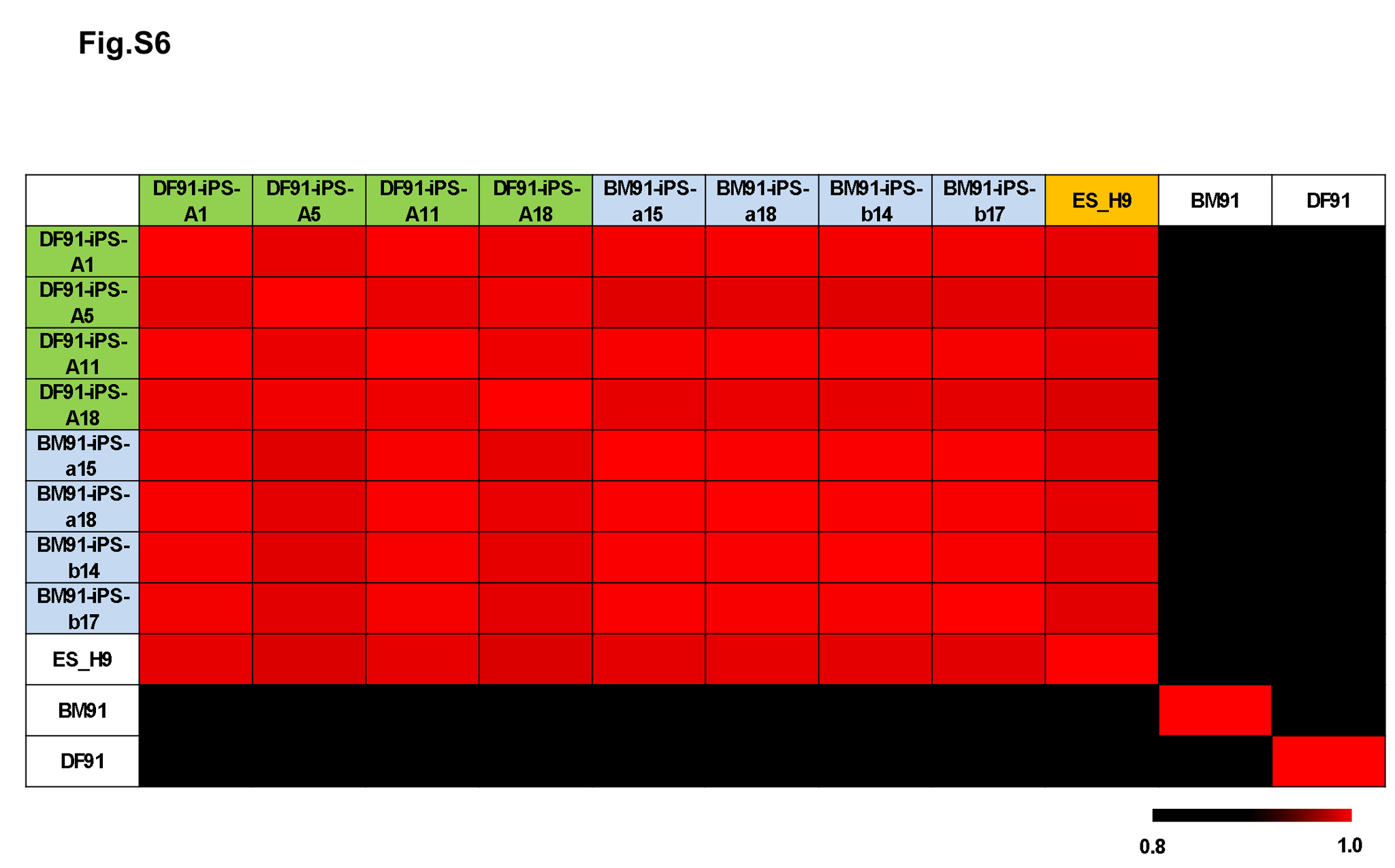

Supplement: Figure S6 — Correlation coefficients between each cell from donor 91 were calculated using gene sets differentially expressed in DFs and BMSCs. (TIF) [file pone.0053771.s006.tif]

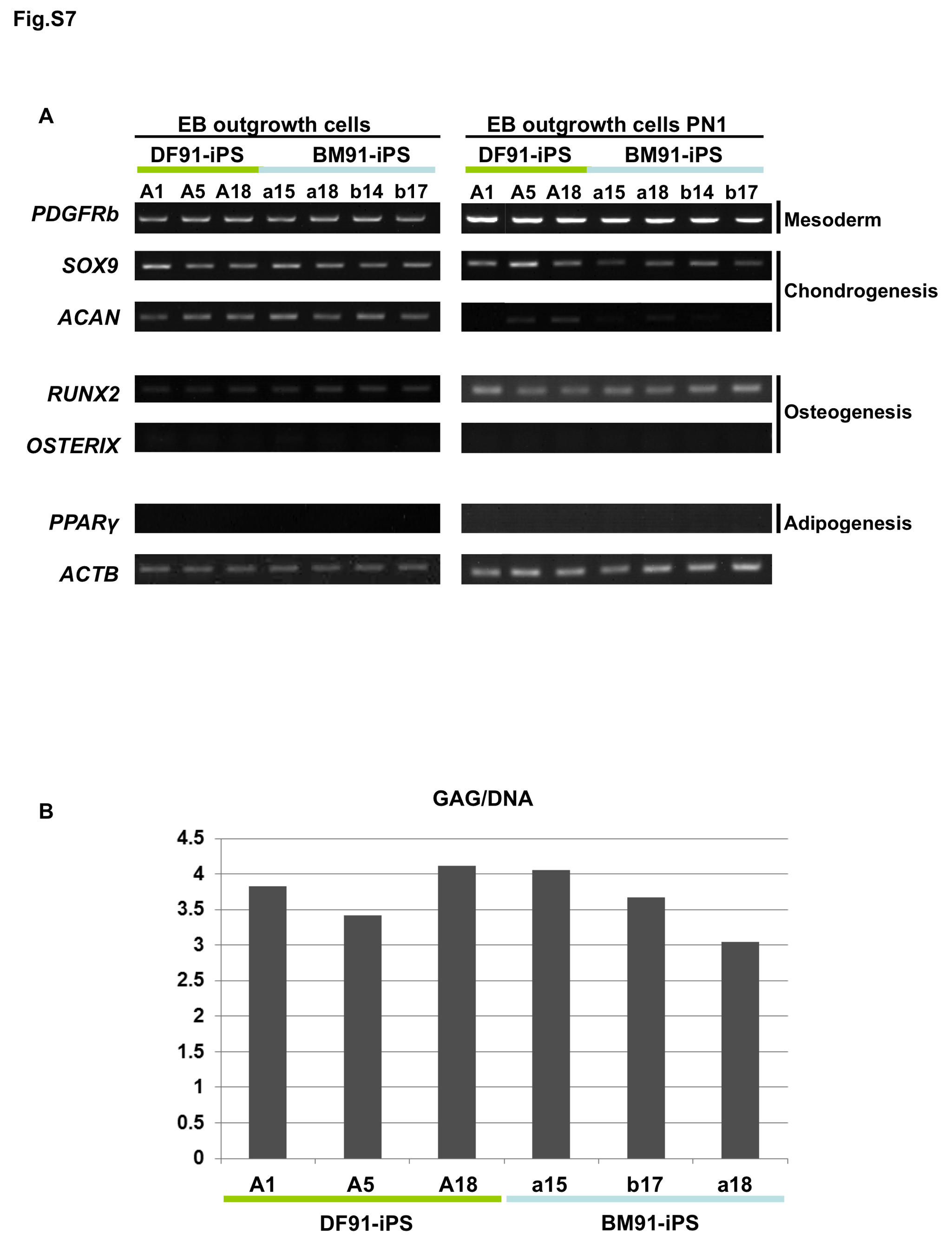

Supplement: Figure S7 — The propensity for differentiation in iPSC clones derived from donor 91 differs regardless of developmental origin. A) The propensity for EB-mediated cell-autonomous differentiation in iPSC clones (donor 91) differs regardless of the developmental origin. B) Induction of chondrogenic differentiation in iPSCs (donor 91). GAG/DNA differed with clones regardless of cell-of-origin. (TIF) [file pone.0053771.s007.tif]

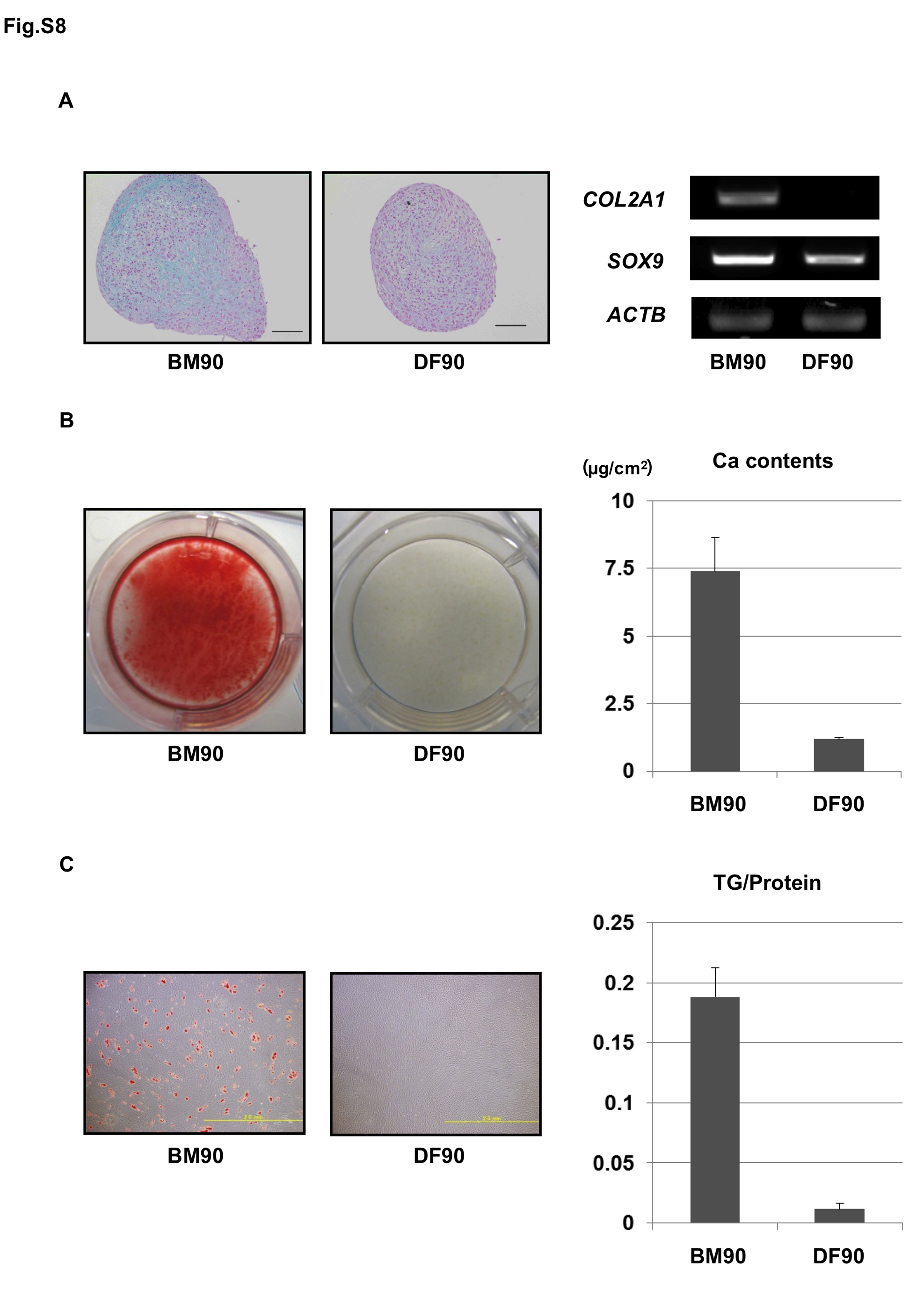

Supplement: Figure S8 — Chondrogenic, osteogenic, and adipogenic differentiation assays with the original BMSCs and DFs. A) Macroscopic views and Alcian blue staining of a section of a pellet (left panel) and expression of chondrogenesis-related genes (SOX9 and COL2) by RT-PCR (right panel). B) Alizarin red staining of osteogenic induction samples (left panel) and calcium contents (right panel). C) Oil-Red-O staining (left panel) and the amount of triglycerides (TG). We used DFs at passage 5–7 and BMs at passage 1–2 for differentiation and confirmed that the DFs used in this study could not differentiate into either chondrocytes, osteoblasts, or adipocytes. Experiments were performed as described previously [21]. (TIF) [file pone.0053771.s008.tif]

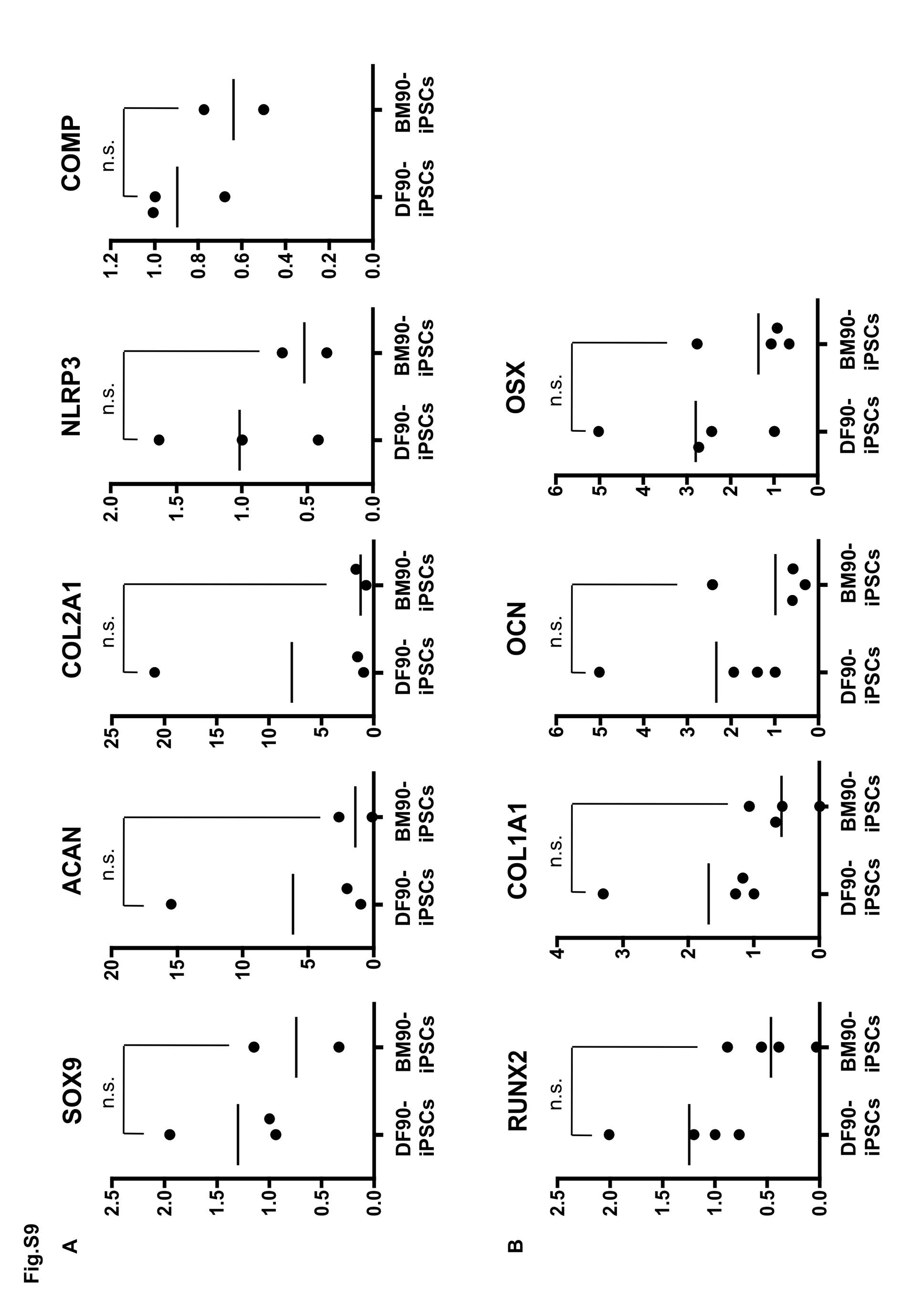

Supplement: Figure S9 — Statistical analyses of differentiation potentials between DF-derived and BM-derived iPSCs. A) Chondrogenic markers. B) Osteogenic markers. Each dot corresponds to each clone. P values are 0.36 (SOX9), 0.49 (ACAN), 0.49 (COL2A1), 0.37 (NLRP3), 0.23 (COMP), 0.052 (RUNX2), 0.11 (COL1A1), 0.24 (OCN), and 0.19 (OSX) (Unpaired t tests). n.s., not significant. (TIF) [file pone.0053771.s009.tif]

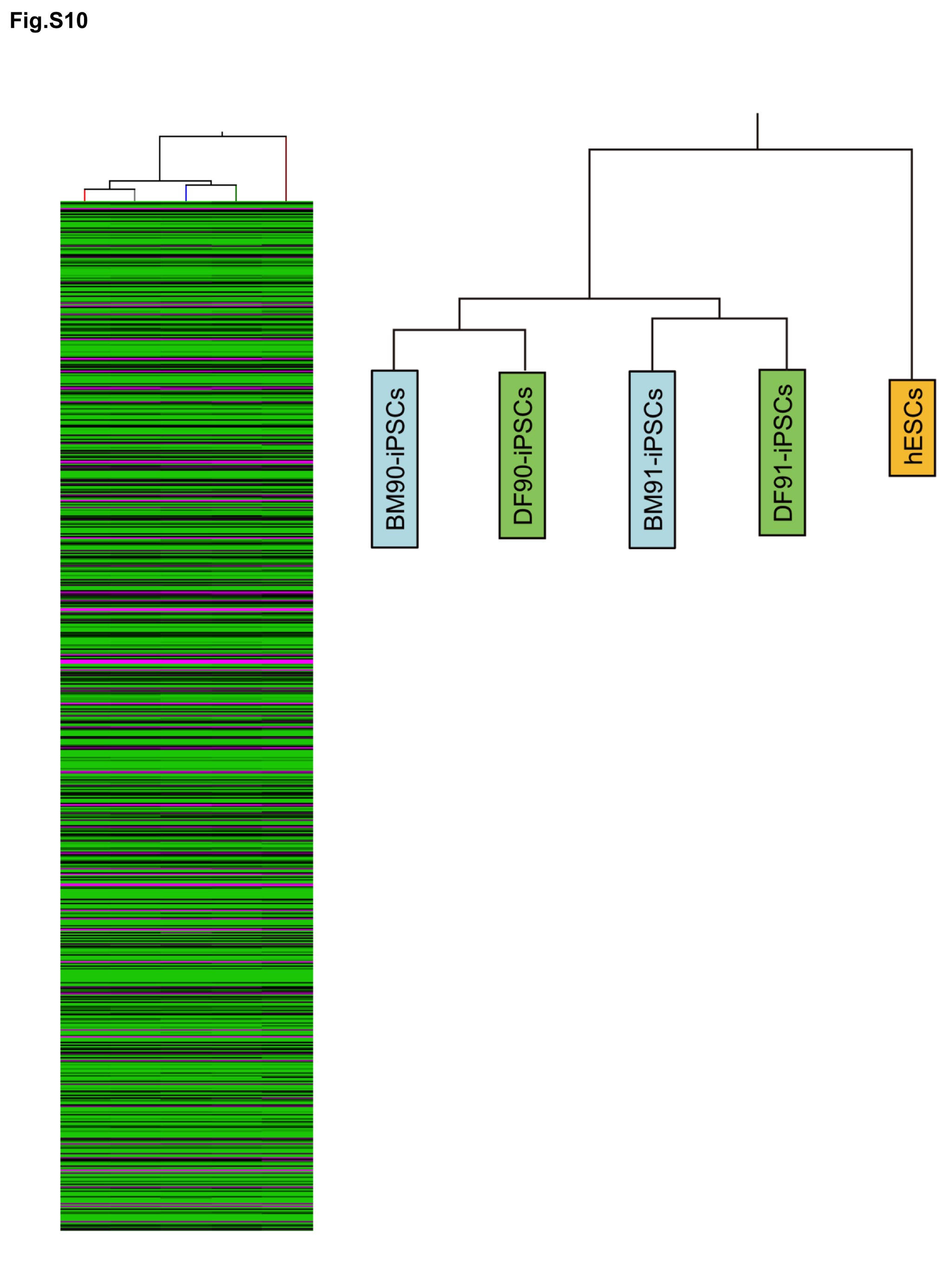

Supplement: Figure S10 — Hierarchical clustering analysis of iPSCs. BM90-iPSCs (average of BM90-iPSC a3, a12, a16, and b6), DF90-iPSCs (average of DF90-iPSC B3 and F2), BM91-iPSCs (average of BM91-iPSC a15, a18, b14, and b17), DF91-iPSCs (average of DF91-iPSC A1, A5, A11, and A18), and hESCs (H9) were subjected to clustering analysis using all gene sets. (TIF) [file pone.0053771.s010.tif]

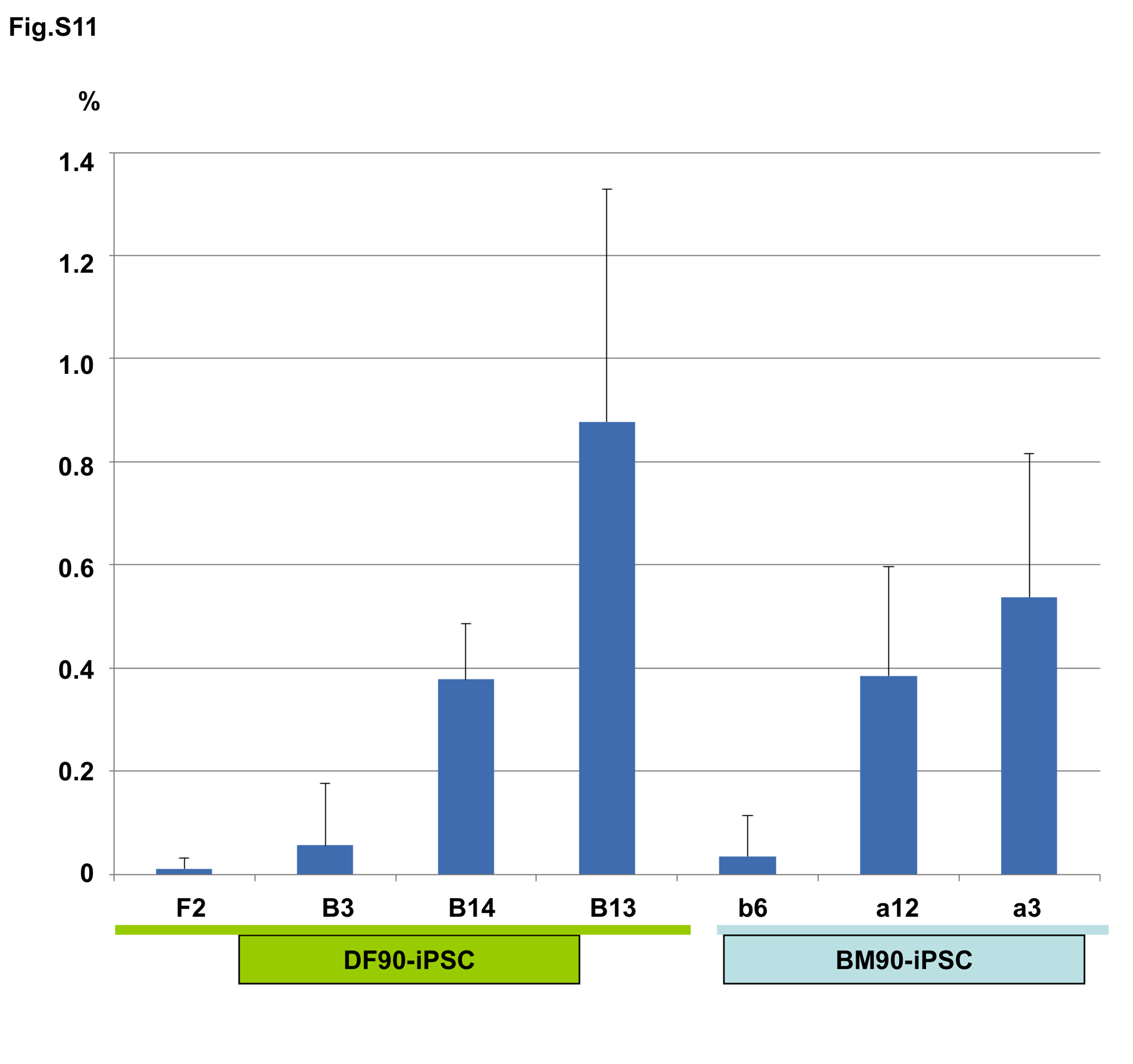

Supplement: Figure S11 — Ratio of cartilage area in teratomas. The cartilage area in teratomas was investigated. Five sections were prepared. Total area and cartilage area detected by Alcian blue staining were calculated using software in BIOREVO (Keyence, Osaka, Japan). (TIF) [file pone.0053771.s011.tif]

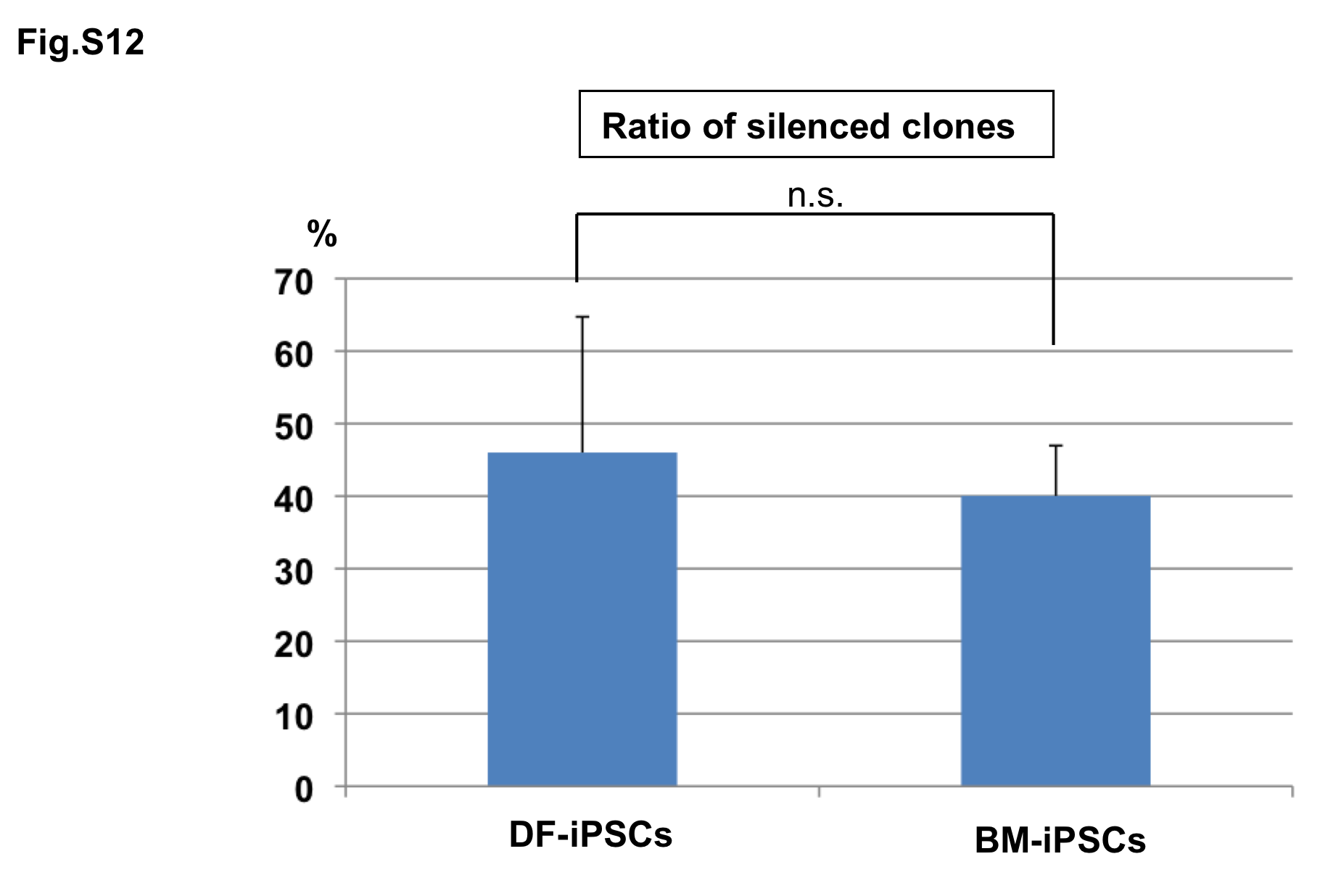

Supplement: Figure S12 — Ratio of transgene-silenced clones. The ratio of clones in which retroviral transgene expression was silenced was less than 1/1000 compared to controls (the value of each transgene 6 days after infection of DF (DF 4F day 6) and 7 days after infection of BM (BM 4F day 7)). (TIF) [file pone.0053771.s012.tif]
